# Sex differences in the genetic architecture of aggressiveness in a sexually dimorphic spider

**DOI:** 10.1002/ece3.5595

**Published:** 2019-08-22

**Authors:** Simona Kralj‐Fišer, Kate L. Laskowski, Francisco Garcia‐Gonzalez

**Affiliations:** ^1^ Evolutionary Zoology Laboratory Institute of Biology Scientific and Research Centre of the Slovenian Academy of Sciences and Arts Ljubljana Slovenia; ^2^ Department of Biology & Ecology of Fishes Leibniz Institute of Freshwater Ecology & Inland Fisheries Berlin Germany; ^3^ Department of Evolution & Ecology University of California Davis Davis CA USA; ^4^ Estación Biológica de Doñana‐CSIC Seville Spain; ^5^ Centre for Evolutionary Biology School of Biological Sciences University of Western Australia Crawley WA Australia

**Keywords:** additive genetic variance, between‐sex genetic correlation, heritability, intralocus sexual conflict, personality, quantitative genetics

## Abstract

Sex differences in the genetic architecture of behavioral traits can offer critical insight into the processes of sex‐specific selection and sexual conflict dynamics. Here, we assess genetic variances and cross‐sex genetic correlations of two personality traits, aggression and activity, in a sexually size‐dimorphic spider, *Nuctenea umbratica*. Using a quantitative genetic approach, we show that both traits are heritable. Males have higher heritability estimates for aggressiveness compared to females, whereas the coefficient of additive genetic variation and evolvability did not differ between the sexes. Furthermore, we found sex differences in the coefficient of residual variance in aggressiveness with females exhibiting higher estimates. In contrast, the quantitative genetic estimates for activity suggest no significant differentiation between males and females. We interpret these results with caution as the estimates of additive genetic variances may be inflated by nonadditive genetic effects. The mean cross‐sex genetic correlations for aggression and activity were 0.5 and 0.6, respectively. Nonetheless, credible intervals of both estimates were broad, implying high uncertainty for these estimates. Future work using larger sample sizes would be needed to draw firmer conclusions on how sexual selection shapes sex differences in the genetic architecture of behavioral traits.

## Graphical abstract

We test for differences between the sexes in genetic variances and examine the cross‐sex genetic correlations, of two important personality traits, aggression and activity in novel environments, in a sexually size dimorphic spider with distinct variation between male and female life history strategies. The present study contributes to expanding our knowledge on the genetic bases of behavioural traits by providing quantitative genetic estimates in aggressiveness and activity in the orb‐weaving spider *N. umbratica*. Our findings further suggest sex differences in the heritability and coefficient of residual variance in aggressiveness, but future work to test cross‐sex genetic correlations in behavioural traits using a larger sample size would be needed to draw firmer conclusions regarding these correlations.

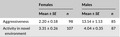

## 1 INTRODUCTION

Consistent individual differences in behavior have been reported for numerous invertebrate and vertebrate taxa (Bell, Hankison, & Laskowski, 2009; Kralj‐Fišer & Schuett, 2014). From a theoretical perspective, variation in personality has been explained by spatio‐temporal variation in selective pressures often generated by state‐dependent positive feedback loops (Sih et al., 2015; Wolf & Weissing, 2010) or negative frequency dependent selection (Dingemanse & Wolf, 2010; Wolf, Van Doorn, Leimar, & Weissing, 2007). However, one major source of variation in selective pressures can be sex‐specific selection (Schuett, Tregenza, & Dall, 2010).

The way that each sex can maximize fitness may differ dramatically. Behavior can play a key role in helping individuals to increase their survival and reproductive success. Indeed, studies have commonly found that males and females differ in the phenotypic expression of personality traits, that is, in average levels of behaviors (Schuett et al., 2010), and in their repeatability, with males being in general more consistent in their behavior than females (Bell et al., 2009). If the sexes share a common genetic architecture for a homologous trait and are exposed to differing selection pressures, such sex‐specific selection may contribute to the maintenance of within‐population variation at the same time that it may result in sex‐specific fitness reductions. Such intralocus sexual conflict may be a potential mechanism explaining the sometimes seemingly maladaptive behavioral responses of individuals (Long & Rice, 2007). A critical first step to understanding the potential role of sex‐specific selection on the evolutionary implications of personality variation is to identify its heritability and underlying genetic architecture, and particularly determining whether it differs across the sexes (Boake et al., 2002; Dingemanse & Réale, 2005).

Given the evolutionary constraints imposed by the common genetic machinery in both sexes for a shared trait, this raises the question of how sex differences in a trait have evolved (Lande, 1980). In theory, an optimal solution would be for each sex to evolve its own optimal set of sex‐ and age‐specific phenotypes (Bonduriansky & Chenoweth, 2009; Lande, 1980, 1987; Rice, 1984). The involved traits are then able to reflect the adaptive divergence in response to selection favoring different optima in the two sexes, resulting in sexual dimorphism (Fairbairn, Blanckenhorn, & Székely, 2007). The genetic architecture of a sexually dimorphic trait, such as behavior, can be studied by the assessment of cross‐sex genetic correlation (*r*_mf_) for the trait. The cross‐sex genetic correlation between homologous male and female traits can be estimated as $r_{\text{mf}}={\text{COV}}_{\text{Amf}}/\sqrt{V_{\text{Af}\;}*V_{\text{Am}}}$, where COV_Amf_ is the additive genetic covariance between the sexes, and *V*_Am_ and *V*_Af_ are additive genetic variances of males and females, respectively (Lande, 1980). When *r*_mf_ is close to unity, the sexes are assumed to have a nearly identical genetic architecture for the trait; close to zero values of *r*_mf_ indicate complete independence in the genetic architecture of the trait between males and females. In the former scenario, a degree of intralocus sexual conflict is expected to persist and (further) evolution of sexual dimorphism should be constrained. In the latter scenario, the evolution of sex‐specific optima should allow for the resolution of sexual conflict and thus the evolution of sexual dimorphism. A cross‐sex genetic correlation between zero and one suggests that some of the genes acting on the shared trait already differ between males and females and indicates a further possibility for the evolution of sexual dimorphism in the trait (Bonduriansky & Chenoweth, 2009; Cox & Calsbeek, 2009).

A recent study in southern field crickets (*Gryllus bimaculatus*) showed higher additive genetic variance for aggression and exploration in males compared to females (Han & Dingemanse, 2017). While *r*_mf_ for aggression was weak and not significantly different from zero suggesting that the genetic architecture of this trait was not constraining its independent evolution in either sex, *r*_mf_ for exploration did not differ from unity suggesting males and females may be constrained if they experience opposing sex‐specific selection on this trait (Han & Dingemanse, 2017). Additionally, a study in Trinidadian guppy, *Poecilia reticulata*, found no evidence for sex‐specific genetic architecture in behaviors related to risk‐taking (White, Houslay, & Wilson, 2019). Researchers are increasingly assessing cross‐sex genetic correlations for morphological and life‐history traits, but little is known about the sex‐specific genetic architecture of “sexually dimorphic” behaviors in general (reviewed in Poissant, Wilson, & Coltman, 2010) and even less in personality traits (e.g., Han & Dingemanse, 2017; Long & Rice, 2007; White et al., 2019).

Furthermore, studies on the heritability of personality traits in invertebrates are scant, and these report mixed results. This is unfortunate, given that invertebrates represent 98% of species in the animal kingdom, and such taxonomic bias can hinder our understanding of the general pattern of personality heritability and thus personality evolution. For instance, moderate heritability has been shown for aggression (e.g., spider *Larinioides sclopetarius*, Kralj‐Fišer & Schneider, 2012), activity (e.g., butterfly *Heliothis armigera*, Colvin & Gatehouse, 1993), and boldness (spider *Agelenopsis pennsylvanica*, Sweeney et al., 2013). Yet, several studies found no support for heritable variation in risk‐taking behavior (pea aphids, *Acyrthosiphon pisum*, Schuett et al., 2011) or boldness (cricket, *Gryllus integer*, Niemelä, Dingemanse, Alioravainen, Vainikka, & Kortet, 2013). Personality traits also differ in their heritability depending on context or ecological conditions (squid *Euprymna tasmanica*, Sinn, Apiolaza, & Moltschaniwskyj, 2006; isopod, *Asellus aquaticus*; Karlsson Green, Eroukhmanoff, Harris, Pettersson, & Svensson, 2016; Australian field cricket *Teleogryllus oceanicus*, Rudin, Simmons, & Tomkins, 2019).

We used a sexually dimorphic model species, the orb‐weaving spider *Nuctenea umbratica*, whose individuals exhibit consistent differences in aggressiveness (repeatability = *r* =0 .78 [95% CI: 0.63, 0.89]) and activity levels (*r* = 0.48 [0.34, 0.63]) (Kralj‐Fišer, Hebets, & Kuntner, 2017), to estimate cross‐sex genetic correlations and narrow‐sense heritabilities of aggressiveness and initial activity in a novel environment. Males are more aggressive in intrasex combats than females (Kralj‐Fišer et al., 2017). However, the sexes show no differences in the mean levels of activity in novel environments (Kralj‐Fišer et al., 2017). As there is no evidence for a genetic association between aggression and activity levels (Kralj‐Fišer et al., 2017), we studied these two traits separately. Based on the previously reported repeatabilities, we expected to find significant heritability of both traits (Lynch & Walsh, 1998). *Nuctenea* spiders are interesting models for studying cross‐sex genetic correlations as females and males exhibit behavioral differences. In particular, females are sit‐and‐wait predators being larger and longer lived than wandering males, which cease foraging after reaching maturity. Aggressiveness toward the same‐sex conspecifics serves males to fight off rivals and enhances access to mates and has been likely shaped by sexual selection. In females, aggressiveness toward same‐sex conspecifics serves a female to defend her territory (web) and thereby foraging patch. Territorial disputes between females are rare (Kralj‐Fišer et al., 2017), probably because overt aggressiveness may have high fitness costs (Kralj‐Fišer & Schneider, 2012). In view of this, we predicted that there would be evidence of sex differences in the genetic underpinning for aggressiveness. On the contrary, we predicted that activity in a novel and potentially risky environment would exhibit similar genetic architecture across the sexes as both sexes should be under similar selection pressures when exposed to unknown stimuli.

## 2 MATERIALS AND METHODS

### 2.1 Study animals

The walnut orb‐weaver spider, *N. umbratica*, is a very common central European species. Females occur all year long, while males appear mainly during summer. During the day, the spiders hide under loose bark or in crevices; in the evening, they build orb‐webs and sit in their center during the night. *Nuctenea umbratica* exhibits sexual‐size dimorphism with females being the larger sex with a sexual dimorphism index (SDI) of 0.6 (Turk, Kuntner, & Kralj‐Fišer, 2018). The cross‐sex genetic correlation (*r*_mf_) for adult mass has been estimated to be 0.92 (Turk et al., 2018).

### 2.2 Animal collection and rearing

We collected subadult *N. umbratica* spiders from their webs on trees and hedgerows along the Ljubljanica riverbank in Ljubljana, Slovenia (46.045093, 14.506048), between May and July 2011. The collected spiders were transferred to the laboratory at the Scientific and Research Centre of the Slovenian Academy of Sciences and Arts, where we kept them individually in 200 ml plastic cups and fed them with fruit flies (*Drosophila* sp.) twice a week. We checked the spiders for molts 5 days a week. Upon sexual maturation, females were transferred into individual plastic frames (36 × 36 × 6 cm), allowing them to construct webs. They were fed two blowflies twice a week (*Calliphora* sp.). The males, which cease web building upon sexual maturation, remained in plastic cups. Throughout the study, we kept the spiders at room temperature under LD 10:14 conditions and misted them with water spray five times a week. We weighed all spiders (accuracy 0.01 mg) before subjecting them to experiments.

### 2.3 Experimental design

#### 2.3.1 Personality tests

The experimental design and data for the parental generation (*N* = 95 spiders; *N* females = 54; *N* males = 41) are published in Kralj‐Fišer et al. (2017). In short, we subjected spiders to two tests: (a) a contest test, which measured an individual's aggressiveness toward a same‐sex conspecific; and (b) a novel environment test, where we measured an individual's activity in a novel environment. Each spider from the parental generation participated in both tests twice, whereas we tested individuals from the offspring generation (*N* = 108 spiders; *N* females = 54; *N* males = 54) in each test once. The order of tests and of observed individuals was randomly established.

##### Aggressiveness toward same‐sex conspecific

In order to assess an individual's level of aggression toward a same‐sex conspecific, we staged intra‐sex contest tests. The individuals were marked; we used a paintbrush to spot a water‐soluble paint on their abdomen. Each individual from parental generation was tested twice in random order, whereas each individual from offspring generation was tested once. Females were tested once as residents in their own web and once as intruders on an unfamiliar web. Males were tested on a random female web (adult males cease web building). In the test, we placed two individuals about five centimeters from each other and recorded agonistic behavior for 20 min. During this time, we noted the frequency of aggressive behaviors. To assess the overall aggressiveness, we scored aggressive behaviors of different intensity as follows: approaching (score = 1), web‐shaking (score = 1), attacking (score = 2), and chasing (score = 3) (e.g., Kralj‐Fišer, Gregorič, Zhang, Li, & Kuntner, 2011; Kralj‐Fišer & Schneider, 2012). We defined “approach” as a movement by one spider toward the other individual, “web‐shaking” as sudden and large amplitude shaking of the web, which spiders usually exhibit when approaching other individuals, “attacking” as a sudden move in the direction of the other individual resulting in a body contact with the opponent, and “chasing” as a running after the (escaping) opponent resulting in a successful attack or escape of the opponent (Kralj‐Fišer et al., 2017). The overall individual's aggressiveness was estimated as the sum of the scores across each trial multiplied by frequencies of observed behaviors, for example, two attacks (2*2) and three chases (3*3) yield a score of 13.

##### Activity in a novel environment

To quantify activity level in novel environment, we carefully placed a test spider into an unfamiliar plastic container (11 × 11 × 6 cm) using a paintbrush. The spider immediately started to walk around the container. We recorded the latency to the first stop, hereafter termed as duration of initial activity in novel environment (e.g., Kralj‐Fišer & Schneider, 2012), with the maximum duration of five minutes. As above, each individual from parental generation was tested twice in random order, whereas each individual from offspring generation was tested once.

#### 2.3.2 Mating

To obtain an offspring generation, we mated all study spiders after the personality tests. Based on the aggressiveness scores from the above experiments, we characterized individuals as aggressive (top third), moderately aggressive (middle third), or nonaggressive (lower third). We mated a subset of spiders (30 females, 30 males) assortatively by aggressiveness levels. This assortative mating was done as part of a second sister study (Kralj‐Fišer et al., 2017). Each female mated with a single male, and each male mated with a single female. We placed a male on a female's web, observed the spiders' behavior for 20 min and then left them together for 24 hr to ensure mating. Thereafter, we kept females and males in their frames and cups, respectively, until natural death. We checked each female's frame for deposited egg cases five times per week. Eighteen females laid at least one viable egg case. The laid egg cases were carefully cut out of the web and stored in a separate container at 25°C until hatching. After the second molt, the spiderlings were separated into individual plastic cups (200 ml) and reared in the same way as outlined above.

#### 2.3.3 Heritability of behaviors

We were able to generate 18 full‐sub families (from 18 males and 18 females). Approximately twenty spiderlings from each family were reared under standardized conditions (as described above) until adulthood. We then assayed three sons and three daughters from each of these families for their behavioral trait values. In total, we included 36 spiders from parental generation (18 females, 18 males) and 108 spiders from the offspring generation (54 females, 54 males). Given the high repeatability in both behaviors in the parental generation (aggressiveness, *r* = 0.78 [95% CI: 0.63, 0.89]; activity, *r* = 0.48 [0.34, 0.63]) (Kralj‐Fišer et al., 2017), we tested the offspring behavior in each assay only once. Male offspring were tested for aggression the same way as their fathers; female offspring were tested as residents on their webs (aggressiveness tested in females as residents and intruders is significantly repeatable (Kralj‐Fišer et al., 2017). The activity in novel environment was measured the same way as in the parental generation. Spiders from both the parental and offspring generations were assayed within the first month after reaching maturity.

### 2.4 Analyses

We calculated estimates of narrow‐sense heritability in aggressiveness and activity using the animal model approach following Wilson et al. (2010). We performed Markov Chain Monte Carlo linear mixed models (using the MCMCglmm package, Hadfield, 2010) analyses in R (version 3.3.1; R Development Core Team, 2013). Animal models use pedigree information to partition the observed phenotypic variance into different genetic and environmental sources and accounts for potential confounding effects (fixed factors). We used the animal model to decompose phenotypic variance (*V*_P_) into additive genetic (*V*_A_), common environment/maternal (*V*_CE/M_), and residual variances (*V*_R_). Our study design does not allow separating maternal from common environment effects since siblings were reared in equal environment until the second molt. We calculated narrow‐sense heritability with 95% credible intervals (CIs) in activity as *h* ^2^ = *V*_A_/(*V*_A_ + *V*_CE/M_ + *V*_R_); however, in aggressiveness we included contest ID (*V*_C_) as a random effect because we recorded aggressiveness scores of both individuals in dyadic contests. We calculated heritability in aggressiveness as *h* ^2^ = *V*_A_/(*V*_A_ + *V*_CE/M_ + *V*_R_ + *V*_C_). When we used sex as a fixed effect in the model, we also included the variance explained by sex into the estimation of the phenotypic variance (De Villemereuil, Morrissey, Nakagawa, & Schielzeth, 2018). Assortative mating can potentially bias the estimates of heritability if not handled correctly. However, we note that assortative mating does not entail bias in the estimation of variance components when using the animal model, especially when phenotypes of all individuals including parents are included in the model (Kruuk, 2004; Walsh & Lynch, 2018). Moreover, when parents and offspring are phenotyped, as in our case, assortative mating increases the precision of the estimates (Michael Morrissey, unpublished).

We also calculated the coefficient of common environment/maternal effect variation as ${\text{CV}}_{\text{CE/M}}=\sqrt{V_{\text{CE/M}}}/\text{mean}$ and the coefficient of residual variation as ${\text{CV}}_{R}=\sqrt{V_{R}}/\text{mean}$. In addition, we calculated two mean‐standardized evolvability measures, the coefficient of additive genetic variation, ${\text{CV}}_{A}=\sqrt{V_{A}}/\text{mean}$, and its square, *I*_A_ = *V*_A_/mean^2^ (Garcia‐Gonzalez, Simmons, Tomkins, Kotiaho, & Evans, 2012; Houle, 1992). CV_A_ and *I*_A_ enable comparing evolvabilities among different traits, sex, and taxa (Garcia‐Gonzalez et al., 2012; Hansen, Pélabon, & Houle, 2011; Houle, 1992). 95% CIs were calculated for all the statistics above. For those parameters whose calculation required the trait mean, namely CV_A_, *I*_A_, CV_CE/M_, CV_R_, phenotypic means obtained from all individuals as well as for each sex separately. We calculated female and male estimates using female and male phenotypic means (Table 1), respectively. Phenotypic means were always calculated using data from both the parental and offspring generations.

**Table 1:** Means and standard errors (*SE*) for aggressiveness scores and activity duration (s) in females and males

	Females		Males	
	Mean ± *SE*	*n*	Mean ± *SE*	*n*
Aggressiveness	2.20 ± 0.18	98	13.14 ± 1.13	85
Activity in novel environment	3.31 ± 0.26	107	4.04 ± 0.35	87

We constructed a pedigree containing every individual included in the experiments (parental and offspring generation). Individuals from the parental generation were field‐collected and their pedigree was unknown. Animal model allows the calculation of kinship among all individuals included in the pedigree to estimate the associated additive genetic variance.

To estimate the effect of sex and assess sex‐specific heritability estimates of aggressiveness and activity we ran three models that differed in fixed and random effect specifications (Table 2, Appendix S1): Model 1 (null model; no fixed effects, random = ~animal + CE/M), model 2 (fixed effect = ~sex, random = ~animal + CE/M), and model 3 (fixed effect = ~sex, random = us(sex):animal + us(sex):CE/M). When modeling aggressiveness, we also added contest ID as a random effect. Models were run using uninformative priors (see Analysis of prior sensitivity in Appendix S1). Models 1 and 2 allow individual variation in intercept and common slopes across all individuals; variance components are not partitioned by sex. We compared deviance information criteria (DIC) obtained by a null model (model 1) to DIC of model 2 to assess the (fixed) effect of sex. When ΔDIC <5, we report no difference between the models; no effect of sex. Model 3 allows sexes to differ in the amount of additive genetic, residual and common environment/maternal variances (allowing to calculate sex‐specific heritability ($h_{f}^{2},\;h_{m}^{2}$). To compare the two sexes, we calculated the mean differences between the female and male for all posterior estimates and obtained the 95% CI for these differences. We calculated cross‐sex genetic correlations (*r*_mf_) as $r_{\text{mf}}={\text{COV}}_{\text{Amf}}/\sqrt{V_{\text{Af}\;}*V_{\text{Am}}}$, where COV_Amf_ is the additive genetic covariance between the sexes, and *V*_Am_ and *V*_Af_ are additive genetic variances of males and females, respectively (Lande, 1980). We checked convergence and mixing properties by visual inspection of the chains and checked the autocorrelation values. We ran Heidelberger and Welch's convergence diagnostics to verify that the number of iterations was adequate for chains to achieve convergence. The R scripts and results are given in Appendix S1 (https://doi.org/10.5061/dryad.5758n3m).

**Table 2:** Results of the quantitative genetic analyses for aggressiveness

M	Fixed factor	Random G‐structure	Fixed effect mean (95% CI)	Sex	*V*_A_ mean (95% CI)	*V*_CE/M_ mean (95% CI)	*V*_R_ mean (95% CI)	*h* ^2^ mean (95% CI)
1	None	animal + CE/M + C	NA	All	60.032 (24.034, 97.622)	1.210 (<0.001, 6.325)	47.231 (25.676, 71.373)	0.364 (0.181, 0.551)
2	Sex	animal + CE/M + C	12.008 (9.098, 14.899)	All	40.520 (16.970, 64.025)	0.934 (<0.001, 4.935)	42.179 (25.106, 60.906)	0.246 (0.123, 0.377)
3	Sex	us(sex):animal + us(sex):CE/M + us(sex):C	11.338 (7.462, 15.276)	Females	2.110 (<0.001, 5.370)	3.931(<0.001, 11.332)	3.324 (1.494, 5.534)	0.040 (<0.001, 0.106)
				Males	77.051 (25.021, 135.715)	12.120 (<0.001, 47.161)	62.763 (28.627, 103.954)	0.259 (0.097, 0.435)

					CV_A_ mean (95% CI)	CV_CE/M_ mean (95% CI)	CV_R_ mean (95% CI)	*I*_A_ mean (95% CI)
1				All	1.041 (0.706, 1.374)	0.091 (0.002, 0.343)	0.928 (0.706, 1.165)	1.113 (0.446, 1.810)
2				All	0.857 (0.596, 1.113)	0.082 (0.002, 0.303)	0.879 (0.687, 1.066)	0.751 (0.315, 1.187)
3				Females	0.594 (<0.001, 1.053)	0.792 (<0.001, 1.530)	0.818 (0.574, 1.082)	0.436 (<0.001, 1.110)
				Males	0.656 (0.414, 0.908)	0.209 (<0.001, 0.523)	0.595 (0.416, 0.781)	0.446 (0.145, 0.786)

Note

DIC1 = 1,942.29; DIC2 = 1,907.237; DIC3 = 1,612.328.

Estimates include posterior mean (95% credible interval = CI) of the fixed effect, additive genetic variance (*V*_A_), common environment/maternal effect variance (*V*_CE/M_), and residual variance (*V*_R_), from the three different models (M) that differed in fixed (fixed factor) and random effect specifications (random G‐structure). We included sex as a fixed factor in models 2 and 3. The random effects were animals' ID, common environment/maternal environments' ID and contests' ID in all models; however, these effects were allowed to vary between sexes in the model 3. We calculated the coefficient of additive genetic variation (CV_A_), coefficient of residual variation (CV_R_), coefficient of common environment/maternal effect variance (CV_CE/M_) and evolvability (*I*_A_) and their 95% CI. In model 1, we calculated heritability (*h* ^2^) as *h* ^2^ = *V*_A_/(*V*_A_ + *V*_CE/M_ + *V*_R_ + *V*_C_), where *V*_C_ stands for variance due to contest ID. When assessing heritability estimates from models 2 and 3, we included the variance explained by the fixed effect into the estimation of the phenotypic variance, *h* ^2^ = *V*_A_/(V_A_ + *V*_CE/M_ + *V*_R_ + *V*_C_ + *V*_f_).

## 3 RESULTS

### 3.1 Aggressiveness

Males were generally more aggressive than females (posterior mean of the effect of males: post. mean = 11.42, 95% credible interval = CI [8.56, 14.19], *p* < .001; see Table 1 for phenotypic means). Aggressiveness was heritable and affected by sex (Table 2). The calculated mean heritability in females was 0.040 (95% CI [<0.001, 0.106]) and 0.259 in males (95% CI [0.097, 0.435]); females had significantly lower heritability estimate than males (mean difference of the female minus males heritability = −0.219, 95% CI [−0.410, −0.041]). However, we found no significant differences between sexes in evolvabilities (CV_A_ or *I*_A_), or the coefficients of common environment/maternal effect variation (CV_CE/M_) (Table 3). Sex difference in the coefficient of residual variance (CV_R_) was marginally nonsignificant, but confidence limits inform that higher female compared to male CV_R_ of aggressiveness levels cannot be ruled out (Table 3).

**Table 3:** Sex differences (female minus male estimate) in the coefficient of additive genetic variation (CV_A_), coefficient of common environment/maternal effect variation (CV_CE/M_) coefficient of residual variation (CV_R_), and evolvability (*I*_A_) for aggressiveness and activity

	CV_A_ mean (95% CI)	CV_CE/M_ mean (95% CI)	CV_R_ mean (95% CI)	*I*_A_ mean (95% CI)
Aggressiveness	−0.062 (−0.683, 0.510)	0.583 (−0.278, 1.464)	0.222 (−0.098, 0.547)	−0.010 (−0.703, 0.803)
Activity	0.002 (−0.051, 0.056)	−0.010 (−0.095, 0.074)	0.028 (−0.002, 0.060)	0.001 (−0.009, 0.010)

The mean additive genetic covariance between males and females estimated by model 3 was 5.576, however, 95% CI overlapped zero [−11.17, 20.839]. The cross‐sex genetic correlation for aggressiveness was nonsignificant (*r*_mf_ = 0.455, 95% CI [−0.911, 1]).

### 3.2 Initial activity in novel environment

Males and females showed no difference in the levels of activity in novel environment (posterior mean of the effect of males = 0.09, 95% CI [−0.04, 0.23], *p* = .179; see phenotypic means in Table 1). Overall, across both sexes, activity levels were moderately heritable and not significantly affected by sex (Table 4). The calculated heritability estimates obtained by model 3 did not differ between the sexes (females: *h^2^* = 0.212 (95% CI [0.035, 0.404]); males: *h^2^* = 0.221 (95% CI [0.059, 0.399])); mean sex difference (female minus male estimate) in *h^2^* = −0.009 (95% CI [−0.249, 0.230]). There was no evidence for differences between the sexes in their estimates of CV_A_, CV_R_, CV_CE/M_, and *I*_A_ (Table 3). The mean additive genetic covariance between males and females estimated was 0.074, 95% CI [−0.133, 0.187]. The cross‐sex genetic correlation for activity was also nonsignificant (*r*_mf_ = 0.603, 95% CI [−0.974, 0.999]).

**Table 4:** Results of the quantitative genetic analyses for activity

M	Fixed factor	Random G‐structure	Fixed effect mean (95% CI)	Sex	*V*_A_ mean (95% CI)	*V*_CE/M_ mean (95% CI)	*V*_R_ mean (95% CI)	*h* ^2^ mean (95% CI)
1	None	animal	NA	All	0.127 (0.062, 0.198)	0.316 (0.095, 0.599)	0.143 (0.102, 0.187)	0.225 (0.095, 0.358)
2	Sex	animal	0.096 (−0.033, 0.224)	All	0.123 (0.057, 0.192)	0.315 (0.099, 0.599)	0.144 (0.103, 0.188)	0.218 (0.092, 0.350)
3	Sex	us(sex):animal + us(sex):CE/M	0.137 (−0.040, 0.307)	Female	0.104 (0.018, 0.191)	0.253 (0.034, 0.551)	0.158 (0.100, 0.222)	0.208 (0.036, 0.35)
				Male	0.147 (0.047, 0.256)	0.420 (0.098, 0.857)	0.137 (0.080, 0.203)	0.219 (0.059, 0.398)

					CV_A_ mean (95% CI)	CV_CE/M_ mean (95% CI)	CV_R_ mean (95% CI)	*I*_A_ mean (95% CI)
1				All	0.097 (0.070, 0.124)	0.152 (0.091, 0.217)	0.104 (0.104, 0.188)	0.010 (0.005, 0.015)
2				All	0.096 (0.068, 0.123)	0.152 (0.093, 0.218)	0.105 (0.090, 0.120)	0.009 (0.004, 0.015)
3				Female	0.095 (0.050, 0.137)	0.145 (0.067, 0.231)	0.119 (0.097, 0.143)	0.009 (0.002, 0.017)
				Male	0.093 (0.058, 0.128)	0.155 (0.084, 0.236)	0.091 (0.071, 0.113)	0.009 (0.003, 0.016)

Note

DIC1 = 356.15; DIC2 = 357.25; DIC3 = 361.25.

Estimates include posterior mean (95% credible interval = CI) of the fixed effect, additive genetic variance (*V*_A_), residual variance (*V*_R_), common environment/maternal effect variance (*V*_CE/M_) from the three different models (M) that differed in fixed (fixed factor) and random effect specifications (random G‐structure). We calculated the coefficient of additive genetic variation (CV_A_), coefficient of residual variation (CV_R_), coefficient of common environment/maternal effect variance (CV_CE/M_) and evolvability (*I*_A_) and their 95% CI. In model 1, we calculated heritability (*h* ^2^) as *h* ^2^ = *V*_A_/(*V*_A_ + *V*_CE/M_ + *V*_R_), whereas we included the variance explained by the fix effect into the estimation of the phenotypic variance when assessing heritability estimates from models 2 and 3: *h* ^2^ = *V*_A_/(*V*_A_ + *V*_CE/M_ + *V*_R_ + *V*_f_).

## 4 DISCUSSION

Despite the increasing interest in research on behavioral variation and consistency, little is known about how the genetic underpinnings of personality traits may differ between the sexes. This is surprising as sex‐specific patterns of genetic variances and heritabilities are key to understanding sex‐specific selection, sexual dimorphism and the evolution and consequences of sexual conflict. Previous research has found that *N. umbratica* males are more aggressive than their female conspecifics but showed no sex differences in mean activity levels (Kralj‐Fišer et al., 2017). Kralj‐Fišer et al.'s (2017) study also showed that individuals consistently vary in both behaviors, but found no correlation between aggressiveness and activity (Kralj‐Fišer et al., 2017). The present study contributes to expanding our knowledge on the genetic bases of behavioral traits by providing quantitative genetic estimates in aggressiveness and activity in the orb‐weaving spider *N. umbratica*. We found that both, aggressiveness and activity, are heritable (Tables 2 and 4) and that the heritability estimates are in line with those found in vertebrates for these traits (reviewed by Dochtermann, Schwab, Anderson Berdal, Dalos, & Royauté, 2019; van Oers & Sinn, 2013). We showed sex differences in the heritability of aggressiveness, being higher in males compared to females. In contrast, the additive genetic coefficient of variation (CV_A_) and evolvability (*I*_A_) did not differ between the sexes (Table 3). The quantitative genetic estimates for activity suggest no significant differentiation between males and females (Table 3). The calculated cross‐sex genetic correlations *r*_mf_ estimates have large credible intervals rendering them imprecise, to obtain robust estimates of *r*_mf_ and 95% CI a larger sample size would be needed.

It has been suggested that fitness traits should have lower heritability compared to nonfitness traits (Mousseau & Roff, 1987). This is because traits related to fitness are expected to have lower additive genetic variation due to strong directional selection (Fisher, 1930; Mousseau & Roff, 1987) or higher residual variances due to, for instance, genic capture and condition dependence (Houle, 1992; Merilä & Sheldon, 1999; Price & Schluter, 1991; Rowe & Houle, 1996). We expect that aggressiveness toward the same‐sex conspecifics strongly relates to fitness components in *Nuctenea* spiders. For example, aggressive males are more likely to gain access to mates increasing their reproductive success, whereas aggressive females may settle in the favorable foraging patches. In both cases, however, overt aggressiveness may decrease survival due to injuries or death. In a previous study, we created high‐density groups of *N. umbratica* females whose composition differed according to aggressiveness types (10 aggressive, 5 aggressive and 5 nonaggressive, or 10 nonaggressive females) (Kralj‐Fišer et al., 2017). However, contrary to the above rationale, we found no relationship between female's variation in aggressiveness toward other females and survival (Kralj‐Fišer et al., 2017). To interpret the higher heritability of aggressiveness in males compared to females, further studies, for example, investigating the relationship between aggressiveness and fitness components, are warranted.

We found no evidence for significant sex differences in genetic variation (CV_A_, *I*_A_) underlying aggressiveness (Table 3) implying that the ability of this trait to respond to selection does not differ between the sexes. However, females show (nonsignificantly) higher coefficient of residual variation (CV_R_) than males. Higher residual variation arguably contributes to the relatively lower estimate of heritability in females. We note that a relatively higher CV_R_ in females may suggest that our scores of females' aggressiveness depend more on the performance of another contestant, whereas males' aggressiveness may be a relatively more intrinsic trait. Sex differences in the amount of nonadditive genetic variance contributing to *V*_R_ (e.g., dominance effect) are unlikely; however, they remain to be addressed by further studies.

We also tested for sex‐specific genetic variances in activity in a novel environment. Our previous data showed that males and females express no differences in activity in novel environment (Kralj‐Fišer et al., 2017), and here, we have documented that heritability and evolvability estimates do not differ between the two sexes (Tables 3 and 4), implying that a potential for evolutionary responses in activity to selection may not vary across males and females. On the other hand, we might have expected to find sex differences in general activity because adult females are rather passive (sit and wait predator), whereas males are wandering actively searching for mates. Thus, adult males might face stronger selection regarding activity than females. It would be worth testing whether sex differences exist in general activity.

The cross‐sex genetic correlations for behavioral traits have been rarely examined, probably because recording behavior on a large enough sample size for quantitative genetic analyses is not easy. We are only aware of two studies. In the cricket *G. bimaculatus*,* r*_mf_ for aggression was significantly lower than 1, whereas *r*_mf_ for exploration was close to unity (Han & Dingemanse, 2017). In guppies (*P. reticulata*), White et al. (2019) found no evidence for sex‐specific genetic architecture in risk‐taking. Our estimates of cross‐sex genetic correlations for aggressiveness and activity heritability estimates for all possible parent–offspring combinations have broad credible intervals and reflect high uncertainty.

The heritability estimates can be inflated by nongenetic effects such as common environment and maternal effects. Our study design does not allow separating maternal from common environment effects since hatchlings (siblings) were reared in an equal environment until the second molt. We included common environment/mother ID as a random effect in the model to account for the resemblance among siblings from the same parents stemming from the same early environment. The common environment/maternal effect explains considerable amount of phenotypic variance in aggressiveness and activity (Tables 2 and 4). These results suggest that maternal effects may play a large role in determining behavioral traits. In spiders, however, little is known about this possibility. Future research on the scope for maternal effects underlying behavioral traits is warranted.

In conclusion, we provide heritability estimates of aggressiveness and activity in *N. umbratica* that fit with estimates of heritability for these traits found in vertebrates, suggesting consistent evolutionary patterns of personality traits across animal taxa. We, however, note that we interpret these estimates with caution, as we cannot rule out that they are inflated by nonadditive genetic effects. Our findings further suggest sex differences in the heritability and coefficient of residual variance in aggressiveness in a sexually dimorphic spider. Future work to test cross‐sex genetic correlations in behavioral traits using a larger sample size would be needed to draw firmer conclusions regarding these correlations.

## CONFLICT OF INTEREST

The authors declare that they have no conflict of interest.

## AUTHOR CONTRIBUTIONS

Simona Kralj Fišer involved in conception and design of the work; data acquisition, analysis, interpretation of results, and writing. Kate Laskowski supported the analysis, interpretation of results, and writing. Francisco Garcia‐Gonzalez supported the analysis, interpretation of results, and writing.

## Data Availability

Data are archived in the Dryad Digital Repository, https://doi.org/10.5061/dryad.5758n3m
