# Supplementary material for: Sex differences in the genetic architecture of aggressiveness in a sexually dimorphic spider
Source: Ecol Evol. 2019 Aug 22;9(18):10758–66. doi: 10.1002/ece3.5595 (PMC6787860; doi:10.1002/ece3.5595)
Supplement: Supplementary file 1 [file ECE3-9-10758-s001.docx]

**Supplementary material**

To estimate heritability in aggressiveness and activity we performed Markov Chain Monte Carlo Linear Mixed Models (using the MCMCglmm package, Hadfield 2010) analyses in R (version 3.3.1, R Core Team 2013).

Supplementary material provides codes and output for three models that differed in fixed (fixed factor) and random effect specifications (random G-structure). We included sex as a fixed factor in models 2 and 3. The random effects were animal’s ID and the ID for the common environment/maternal environment in all models, For aggressiveness we also included contest’s ID as a random factor as this trait was measured in dyads across the individuals measured. These effects were allowed to vary (to have different variances) between sexes in the model 3. Each model was run using two different (uninformative) priors.

We calculated posterior mean (and the 95% credible interval = CI ) of the fixed effect, additive genetic variance (*V_A_*), common environment/maternal effect variance (*V_CE/M_*), and residual variance (*V_R_*). In addition we calculated mean-standardized evolvabilities (coefficient of additive genetic variation (*CV_A_*) and (*I_A_*)), the coefficient of residual variation (*CV_R_*), and the coefficient of common environment/maternal effect variance (*CV_CE/M_*) plus their 95% CI.

Aggressiveness: In model 1 we calculated heritability (*h^2^*) as $h^{2}=\frac{V_{A}}{(V_{A}+{V_{CE/M}+ V}_{R}+V_{C})}$, where *V_C_* stands for variance due to contest ID. When assessing heritability estimates from models 2 and 3 we included the variance explained by the fixed effect into the estimation of the phenotypic variance; $h^{2}=\frac{V_{A}}{(V_{A}+{V_{CE/M}+ V}_{R}+V_{C}+ Vf)}$.

Activity: In model 1 we calculated heritability (*h^2^*) as $h^{2}=\frac{V_{A}}{(V_{A}+{V_{CE/M}+ V}_{R})}$. In models 2 and 3 we calculated heritability as $h^{2}=\frac{V_{A}}{(V_{A}+{V_{CE/M}+ V}_{R}+ Vf)}$

In the second part we provide data.

Table of Contents

[AGGRESSIVENESS 4](#_Toc15634889)

[## Model 1 (fixed factor = none, random = animal + common environment/maternal effect + contest ID) 4](#_Toc15634890)

[Prior 1 4](#_Toc15634891)

[Prior 2 7](#_Toc15634892)

[## Model 2 (fixed factor = sex, random = animal + common environment/maternal effect + contest ID) 10](#_Toc15634893)

[Prior 1 10](#_Toc15634894)

[Prior 2 13](#_Toc15634895)

[## Model 3 (fixed factor = none, random=~us(SEX):animal + ~us(SEX):common environment/maternal effect + ~us(SEX):contest ID) 16](#_Toc15634896)

[Prior 1 16](#_Toc15634897)

[Prior 2 22](#_Toc15634898)

[ACTIVITY 28](#_Toc15634900)

[## Model 1 (fixed factor = none, random = animal + common environment/maternal effect) 28](#_Toc15634901)

[Prior 1 28](#_Toc15634902)

[Prior 2 31](#_Toc15634903)

[## Model 2 (fixed factor = sex, random = animal + common environment/maternal effect) 34](#_Toc15634904)

[Prior 1 34](#_Toc15634905)

[Prior 2 36](#_Toc15634906)

[## Model 3 (fixed factor = sex, random = us(SEX):animal + us(SEX):common environment/maternal effect) 40](#_Toc15634907)

[Prior 1 40](#_Toc15634908)

[Prior 2 46](#_Toc15634909)

[Data 52](#_Toc15634910)

[Aggression 52](#_Toc15634911)

[Activity 63](#_Toc15634912)

# AGGRESSIVENESS

## ## Model 1 (fixed factor = none, random = animal + common environment/maternal effect + contest ID)

### Prior 1

**prior1<-list(G = list(G1 = list(V = 1, nu = 0.002), G2 = list(V = 1, nu = 0.002), G3 = list(V = 1, nu = 0.002)), R = list(V = 1, nu = 0.002))**

**model1 <- MCMCglmm(AGG ~ 1, random = ~animal + PE + Idf, pedigree = AggPed,data = Data4A, nitt = 5000000, thin = 100, burnin = 150000, prior = prior1, verbose = FALSE)**

Summary

Iterations = 150001:4999901

Thinning interval = 100

Sample size = 48500

DIC: 1942.29

G-structure: ~animal

post.mean l-95% CI u-95% CI eff.samp

animal 60.03 24.03 97.62 48500 ***# V_A_***

~PE

post.mean l-95% CI u-95% CI eff.samp ***# V_CE/M_***

PE 1.21 0.000159 6.325 42843

~Idf

post.mean l-95% CI u-95% CI eff.samp ***# V_C_***

Idf 54.76 29.57 83.17 48500

R-structure: ~units

post.mean l-95% CI u-95% CI eff.samp ***# V_R_***

units 47.23 25.65 71.37 48500

Location effects: AGG ~ 1

post.mean l-95% CI u-95% CI eff.samp pMCMC

(Intercept) 6.886 4.586 9.149 48500 <2e-05 ***

---

Signif. codes: 0 ‘***’ 0.001 ‘**’ 0.01 ‘*’ 0.05 ‘.’ 0.1 ‘ ’ 1

> plot(model1$Sol)

> plot(model1$VCV)

> autocorr.diag(model1$Sol)

(Intercept)

Lag 0 1.0000000000

Lag 100 0.0020367136

Lag 500 0.0056297219

Lag 1000 0.0069718098

Lag 5000 -0.0006479138

> autocorr.diag(model1$VCV)

animal PE Idf units

Lag 0 1.000000000 1.000000000 1.000000000 1.000000000

Lag 100 0.004693029 0.049596200 0.002801076 0.006140161

Lag 500 -0.003988453 0.001888816 -0.001606295 -0.001760823

Lag 1000 -0.004925383 0.012858942 -0.001978215 0.003243930

Lag 5000 -0.004038559 -0.005572553 -0.003610646 0.003015379

> effectiveSize(model1$Sol)

(Intercept)

48500

> effectiveSize(model1$VCV)

animal PE Idf units

48500 42843 48500 48500

> heidel.diag(model1$VCV)

Stationarity start p-value

test iteration

animal passed 1 0.749

PE passed 1 0.123

Idf passed 1 0.917

units passed 1 0.483

Halfwidth Mean Halfwidth

test

animal passed 60.03 0.170

PE passed 1.21 0.032

Idf passed 54.76 0.122

units passed 47.23 0.107

> HPDinterval(model1$VCV)

lower upper

animal 2.403427e+01 97.621883

PE 1.590386e-04 6.324726

Idf 2.957054e+01 83.171845

units 2.564633e+01 71.373162

attr(,"Probability")

[1] 0.95

> vA1 <- model1$VCV[,'animal']

> vR1 <- model1$VCV[,'units']

> vPE1 <- model1$VCV[,'PE']

> vdf1 <- model1$VCV[,'Idf']

> mean(vA1)

[1] 60.03204

> HPDinterval(vA1)

lower upper

var1 24.03427 97.62188

attr(,"Probability")

[1] 0.95

>

> mean(vR1)

[1] 47.23093

> HPDinterval(vR1)

lower upper

var1 25.64633 71.37316

attr(,"Probability")

[1] 0.95

>

>

> mean(vPE1)

[1] 1.209578

> HPDinterval(vPE1)

lower upper

var1 0.0001590386 6.324726

attr(,"Probability")

[1] 0.95

>

> mean(vdf1)

[1] 54.75886

> HPDinterval(vdf1)

lower upper

var1 29.57054 83.17185

attr(,"Probability")

[1] 0.95

>

> herit1<- vA1/(vA1 + vR1 + vPE1 + vdf1) **#** $\boldsymbol{h}^{\boldsymbol{2}}\mathbf{=}\frac{\boldsymbol{V}_{\boldsymbol{A}}}{\left( \boldsymbol{V}_{\boldsymbol{A}}\mathbf{+}{\boldsymbol{V}_{\boldsymbol{CE/M}}\boldsymbol{+ V}}_{\boldsymbol{R}}\mathbf{+}\boldsymbol{V}_{\boldsymbol{C}} \right)}$

> mean(herit1)

[1] 0.3641134 **# mean heritability**

> HPDinterval(herit1)

lower upper

var1 0.1813421 0.5512268 **#95% CI heritability**

attr(,"Probability")

[1] 0.95

> CVA1<- sqrt(model1$VCV[, "animal"])/7.343

> mean(CVA1) ***# CV_A_***

[1] 1.041356

> HPDinterval(CVA1)

lower upper

var1 0.7060844 1.374075

attr(,"Probability")

[1] 0.95

>

> I1<- model1$VCV[, "animal"]/53.920 ***# I***

> mean(I1)

[1] 1.113354

> HPDinterval(I1)

lower upper

var1 0.4457394 1.810495

attr(,"Probability")

[1] 0.95

>

> CVR1<- sqrt(model1$VCV[, "units"])/7.343 ***# CV_R_***

> mean(CVR1)

[1] 0.9284869

> HPDinterval(CVR1)

lower upper

var1 0.705802 1.165112

attr(,"Probability")

[1] 0.95

> CVPE1<- sqrt(model1$VCV[, "PE"])/7.343 ***# CV_CE/M_***

> mean(CVPE1)

[1] 0.09107045

> HPDinterval(CVPE1)

lower upper

var1 0.001777444 0.3425146

attr(,"Probability")

[1] 0.95

________________________________________________________

### Prior 2

**prior1.1<-list(G=list(G1=list(V=matrix(p.var*0.5),n=1), G2 = list(V = 1, nu = 0.002), G3 = list(V = 1, nu = 0.002)),R=list(V=matrix(p.var*0.5),n=1))**

**model1 <- MCMCglmm(AGG ~ 1, random = ~animal + PE + Idf, pedigree = AggPed,data = Data4A, nitt = 5000000, thin = 100, burnin = 150000, prior = prior1.1, verbose = FALSE)**

Summary

Iterations = 150001:4999901

Thinning interval = 100

Sample size = 48500

DIC: 1944.989

G-structure: ~animal

post.mean l-95% CI u-95% CI eff.samp

animal 60.51 27.07 96.53 48500

~PE

post.mean l-95% CI u-95% CI eff.samp

PE 1.232 0.0001525 6.506 44073

~Idf

post.mean l-95% CI u-95% CI eff.samp

Idf 54.08 28.09 81.28 48500

R-structure: ~units

post.mean l-95% CI u-95% CI eff.samp

units 47.62 26.17 70.24 48500

Location effects: AGG ~ 1

post.mean l-95% CI u-95% CI eff.samp pMCMC

(Intercept) 6.897 4.641 9.144 44535 <2e-05 ***

---

Signif. codes: 0 ‘***’ 0.001 ‘**’ 0.01 ‘*’ 0.05 ‘.’ 0.1 ‘ ’ 1

> plot(model1$Sol)

> plot(model1$VCV)

> autocorr.diag(model1$Sol)

(Intercept)

Lag 0 1.000000000

Lag 100 0.002605384

Lag 500 0.001259294

Lag 1000 0.016727333

Lag 5000 0.001193842

> autocorr.diag(model1$VCV)

animal PE Idf units

Lag 0 1.000000000 1.000000000 1.0000000000 1.0000000000

Lag 100 0.004364943 0.042628499 0.0001922233 -0.0005067018

Lag 500 -0.004024562 0.004460415 -0.0007960150 -0.0068303138

Lag 1000 -0.001987846 -0.007004613 0.0028433595 0.0001116302

Lag 5000 0.004373965 -0.003393105 0.0020937924 0.0070711876

> effectiveSize(model1$Sol)

(Intercept)

44534.83

> effectiveSize(model1$VCV)

animal PE Idf units

48500.0 44072.7 48500.0 48500.0

> heidel.diag(model1$VCV)

Stationarity start p-value

test iteration

animal passed 1 0.389

PE passed 1 0.841

Idf passed 1 0.238

units passed 1 0.852

Halfwidth Mean Halfwidth

test

animal passed 60.51 0.1612

PE passed 1.23 0.0318

Idf passed 54.08 0.1210

units passed 47.62 0.1024

> HPDinterval(model1$VCV)

lower upper

animal 2.706616e+01 96.526215

PE 1.525328e-04 6.506263

Idf 2.808870e+01 81.276759

units 2.617255e+01 70.244170

attr(,"Probability")

[1] 0.95

> vA1 <- model1$VCV[,'animal']

> vR1 <- model1$VCV[,'units']

> vPE1 <- model1$VCV[,'PE']

> vdf1 <- model1$VCV[,'Idf']

>

> mean(vA1)

[1] 60.51321

> HPDinterval(vA1)

lower upper

var1 27.06616 96.52621

attr(,"Probability")

[1] 0.95

>

> mean(vR1)

[1] 47.61696

> HPDinterval(vR1)

lower upper

var1 26.17255 70.24417

attr(,"Probability")

[1] 0.95

>

>

> mean(vPE1)

[1] 1.232413

> HPDinterval(vPE1)

lower upper

var1 0.0001525328 6.506263

attr(,"Probability")

[1] 0.95

>

> mean(vdf1)

[1] 54.08456

> HPDinterval(vdf1)

lower upper

var1 28.0887 81.27676

attr(,"Probability")

[1] 0.95

>

> herit1<- vA1/(vA1 + vR1 + vPE1 + vdf1)

> mean(herit1)

[1] 0.3671091

> HPDinterval(herit1)

lower upper

var1 0.1914839 0.5395074

attr(,"Probability")

[1] 0.95

>

> CVA1<- sqrt(model1$VCV[, "animal"])/7.343

> mean(CVA1)

[1] 1.047311

> HPDinterval(CVA1)

lower upper

var1 0.7391335 1.361557

attr(,"Probability")

[1] 0.95

>

> I1<- model1$VCV[, "animal"]/53.920

> mean(I1)

[1] 1.122278

> HPDinterval(I1)

lower upper

var1 0.5019689 1.790175

attr(,"Probability")

[1] 0.95

>

> CVR1<- sqrt(model1$VCV[, "units"])/7.343

> mean(CVR1)

[1] 0.9329917

> HPDinterval(CVR1)

lower upper

var1 0.7205624 1.159509

attr(,"Probability")

[1] 0.95

>

>

> CVPE1<- sqrt(model1$VCV[, "PE"])/7.343

> mean(CVPE1)

[1] 0.09237018

> HPDinterval(CVPE1)

lower upper

var1 0.001754513 0.3473812

attr(,"Probability")

[1] 0.95

XXXXXXXXXXXXXXXXXXXXXXXXXXXXXXXXXXXXXXXXXXXXXXXXXXXXXXXXX

## ## Model 2 (fixed factor = sex, random = animal + common environment/maternal effect + contest ID)

### Prior 1

**prior1<-list(G = list(G1 = list(V = 1, nu = 0.002), G2 = list(V = 1, nu = 0.002), G3 = list(V = 1, nu = 0.002)), R = list(V = 1, nu = 0.002))**

**model2 <- MCMCglmm(AGG ~ SEX, random = ~animal + PE + Idf, pedigree = AggPed,data = Data4A, nitt = 5000000, thin = 100, burnin = 150000, prior = prior1, verbose = FALSE)**

Summary

Iterations = 150001:4999901

Thinning interval = 100

Sample size = 48500

DIC: 1907.237

G-structure: ~animal

post.mean l-95% CI u-95% CI eff.samp

animal 40.52 16.97 64.02 48500

~PE

post.mean l-95% CI u-95% CI eff.samp

PE 0.9343 0.0001358 4.935 42587

~Idf

post.mean l-95% CI u-95% CI eff.samp

Idf 43.71 23.74 65.17 48500

R-structure: ~units

post.mean l-95% CI u-95% CI eff.samp

units 42.18 25.11 60.91 46703

Location effects: AGG ~ SEX

post.mean l-95% CI u-95% CI eff.samp pMCMC

(Intercept) 1.6152 -0.7162 3.9563 47370 0.176

SEX2 12.0078 9.0978 14.8994 48500 <2e-05 *** **# fixed effect**

---

Signif. codes: 0 ‘***’ 0.001 ‘**’ 0.01 ‘*’ 0.05 ‘.’ 0.1 ‘ ’ 1

> plot(model2$Sol)

> plot(model2$VCV)

> autocorr.diag(model2$Sol)

(Intercept) SEX2

Lag 0 1.0000000000 1.0000000000

Lag 100 0.0050537116 -0.0032389301

Lag 500 0.0012612912 -0.0014605030

Lag 1000 -0.0015274028 -0.0043837581

Lag 5000 0.0006939502 -0.0007739482

> autocorr.diag(model2$VCV)

animal PE Idf units

Lag 0 1.0000000000 1.000000000 1.000000000 1.000000000

Lag 100 -0.0009841596 0.056513360 0.005062810 0.006331155

Lag 500 -0.0012011018 -0.004259023 -0.004747304 -0.002602630

Lag 1000 0.0055126733 -0.001126968 -0.002616672 0.001221060

Lag 5000 -0.0096073453 0.001824498 0.007302284 -0.001006147

> effectiveSize(model2$Sol)

(Intercept) SEX2

47369.76 48500.00

> effectiveSize(model2$VCV)

animal PE Idf units

48500.00 42587.01 48500.00 46703.46

> heidel.diag(model2$VCV)

Stationarity start p-value

test iteration

animal passed 1 0.142

PE passed 1 0.597

Idf passed 1 0.356

units passed 1 0.698

Halfwidth Mean Halfwidth

test

animal passed 40.520 0.1084

PE passed 0.934 0.0233

Idf passed 43.712 0.0940

units passed 42.179 0.0852

> HPDinterval(model2$VCV)

lower upper

animal 1.696972e+01 64.024812

PE 1.358164e-04 4.934597

Idf 2.373912e+01 65.173263

units 2.510571e+01 60.906465

attr(,"Probability")

[1] 0.95

> vA2 <- model2$VCV[,'animal']

> vR2 <- model2$VCV[,'units']

> vPE2 <- model2$VCV[,'PE']

> vIdf2 <- model2$VCV[,'Idf']

>

> mean(vA2)

[1] 40.52025

> HPDinterval(vA2)

lower upper

var1 16.96972 64.02481

attr(,"Probability")

[1] 0.95

>

> mean(vR2)

[1] 42.17947

> HPDinterval(vR2)

lower upper

var1 25.10571 60.90647

attr(,"Probability")

[1] 0.95

>

> mean(vPE2)

[1] 0.9343052

> HPDinterval(vPE2)

lower upper

var1 0.0001358164 4.934597

attr(,"Probability")

[1] 0.95

>

> mean(vIdf2)

[1] 43.71155

> HPDinterval(vIdf2)

lower upper

var1 23.73912 65.17326

attr(,"Probability")

[1] 0.95

>

> Vf2 <- sapply(1:nrow(model2[["Sol"]]), function(i) {var(predict(model2, it = i)) }) ***#V_f_***

> mean(Vf2)

[1] 36.42633

>

>

> herit2<- vA2/(vA2 + vR2 + vPE2 + vIdf2+ Vf2) **#** $\boldsymbol{h}^{\boldsymbol{2}}\mathbf{=}\frac{\boldsymbol{V}_{\boldsymbol{A}}}{\left( \boldsymbol{V}_{\boldsymbol{A}}\mathbf{+}{\boldsymbol{V}_{\boldsymbol{CE/M}}\boldsymbol{+ V}}_{\boldsymbol{R}}\mathbf{+}\boldsymbol{V}_{\boldsymbol{C}}\boldsymbol{+}\boldsymbol{V}_{\boldsymbol{f}} \right)}$

> mean(herit2)

[1] 0.2462916

> HPDinterval(herit2)

lower upper

var1 0.1225613 0.3774555

attr(,"Probability")

[1] 0.95

> CVA2<- sqrt(model2$VCV[, "animal"])/7.343

> mean(CVA2)

[1] 0.8568678

> HPDinterval(CVA2)

lower upper

var1 0.5955457 1.112704

attr(,"Probability")

[1] 0.95

>

> I2<- model2$VCV[, "animal"]/53.920

> mean(I2)

[1] 0.7514884

> HPDinterval(I2)

lower upper

var1 0.3147204 1.187404

attr(,"Probability")

[1] 0.95

>

> CVR2<- sqrt(model2$VCV[, "units"])/7.343

> mean(CVR2)

[1] 0.8790702

> HPDinterval(CVR2)

lower upper

var1 0.6869449 1.066389

attr(,"Probability")

[1] 0.95

>

> CVPE2<- sqrt(model2$VCV[, "PE"])/7.343

> mean(CVPE2)

[1] 0.08204482

> HPDinterval(CVPE2)

lower upper

var1 0.001854018 0.3025898

attr(,"Probability")

[1] 0.95

__________________________________________________________________________________

### Prior 2

**prior1.1<-list(G=list(G1=list(V=matrix(p.var*0.5),n=1), G2 = list(V = 1, nu = 0.002), G3 = list(V = 1, nu = 0.002)),R=list(V=matrix(p.var*0.5),n=1))**

**model2 <- MCMCglmm(AGG ~ SEX, random = ~animal + PE + Idf, pedigree = AggPed,data = Data4A, nitt = 5000000, thin = 100, burnin = 150000, prior = prior1.1, verbose = FALSE)**

Summary

Iterations = 150001:4999901

Thinning interval = 100

Sample size = 48500

DIC: 1908.165

G-structure: ~animal

post.mean l-95% CI u-95% CI eff.samp

animal 41.77 20.23 64.62 48500

~PE

post.mean l-95% CI u-95% CI eff.samp

PE 0.9155 0.0001663 4.733 43114

~Idf

post.mean l-95% CI u-95% CI eff.samp

Idf 43.32 23.28 64.5 48500

R-structure: ~units

post.mean l-95% CI u-95% CI eff.samp

units 42.27 25.47 60.1 49592

Location effects: AGG ~ SEX

post.mean l-95% CI u-95% CI eff.samp pMCMC

(Intercept) 1.6027 -0.7573 3.9792 48500 0.18

SEX2 12.0146 9.1439 14.9692 48500 <2e-05 ***

---

Signif. codes: 0 ‘***’ 0.001 ‘**’ 0.01 ‘*’ 0.05 ‘.’ 0.1 ‘ ’ 1

> plot(model2$Sol)

> plot(model2$VCV)

> autocorr.diag(model2$Sol)

(Intercept) SEX2

Lag 0 1.000000000 1.000000000

Lag 100 -0.002757170 -0.003564147

Lag 500 -0.001808434 0.002746226

Lag 1000 0.001337962 0.003678009

Lag 5000 0.007297995 0.004926340

> autocorr.diag(model2$VCV)

animal PE Idf units

Lag 0 1.000000000 1.0000000000 1.000000000 1.000000000

Lag 100 -0.002598252 0.0587805993 -0.005481010 -0.011145929

Lag 500 -0.004378493 0.0034200724 -0.003228808 -0.010257776

Lag 1000 -0.001234366 -0.0034923547 0.004091386 -0.003185590

Lag 5000 0.001699559 0.0003259065 0.003216878 -0.001841633

> effectiveSize(model2$Sol)

(Intercept) SEX2

48500 48500

> effectiveSize(model2$VCV)

animal PE Idf units

48500.00 43113.94 48500.00 49592.32

> heidel.diag(model2$VCV)

Stationarity start p-value

test iteration

animal passed 1 0.0616

PE passed 1 0.4870

Idf passed 1 0.7436

units passed 4851 0.1320

Halfwidth Mean Halfwidth

test

animal passed 41.772 0.1033

PE passed 0.915 0.0234

Idf passed 43.324 0.0937

units passed 42.244 0.0838

> HPDinterval(model2$VCV)

lower upper

animal 2.022582e+01 64.615355

PE 1.663351e-04 4.732697

Idf 2.327625e+01 64.496164

units 2.546954e+01 60.102926

attr(,"Probability")

[1] 0.95

> vA2 <- model2$VCV[,'animal']

> vR2 <- model2$VCV[,'units']

> vPE2 <- model2$VCV[,'PE']

> vIdf2 <- model2$VCV[,'Idf']

>

> mean(vA2)

[1] 41.772

> HPDinterval(vA2)

lower upper

var1 20.22582 64.61535

attr(,"Probability")

[1] 0.95

>

> mean(vR2)

[1] 42.27372

> HPDinterval(vR2)

lower upper

var1 25.46954 60.10293

attr(,"Probability")

[1] 0.95

>

> mean(vPE2)

[1] 0.915467

> HPDinterval(vPE2)

lower upper

var1 0.0001663351 4.732697

attr(,"Probability")

[1] 0.95

>

> mean(vIdf2)

[1] 43.32443

> HPDinterval(vIdf2)

lower upper

var1 23.27625 64.49616

attr(,"Probability")

[1] 0.95

>

> Vf2 <- sapply(1:nrow(model2[["Sol"]]), function(i) {var(predict(model2, it = i)) })

>

> mean(Vf2)

[1] 36.46467

>

>

> herit2<- vA2/(vA2 + vR2 + vPE2 + vIdf2+ Vf2)

> mean(herit2)

[1] 0.2526387

> HPDinterval(herit2)

lower upper

var1 0.1325157 0.3705125

attr(,"Probability")

[1] 0.95

>

>

>

> CVA2<- sqrt(model2$VCV[, "animal"])/7.343

> mean(CVA2)

[1] 0.8716522

> HPDinterval(CVA2)

lower upper

var1 0.6336168 1.110798

attr(,"Probability")

[1] 0.95

>

> I2<- model2$VCV[, "animal"]/53.920

> mean(I2)

[1] 0.7747033

> HPDinterval(I2)

lower upper

var1 0.375108 1.198356

attr(,"Probability")

[1] 0.95

>

> CVR2<- sqrt(model2$VCV[, "units"])/7.343

> mean(CVR2)

[1] 0.8804912

> HPDinterval(CVR2)

lower upper

var1 0.7015327 1.066581

attr(,"Probability")

[1] 0.95

>

> CVPE2<- sqrt(model2$VCV[, "PE"])/7.343

> mean(CVPE2)

[1] 0.08087867

> HPDinterval(CVPE2)

lower upper

var1 0.00175638 0.2962653

attr(,"Probability")

[1] 0.95

## ## Model 3 (fixed factor = none, random=~us(SEX):animal + ~us(SEX):common environment/maternal effect + ~us(SEX):contest ID)

### Prior 1

**prior2.6 <- list(R=list(V=diag(2), nu=2), G=list(G1=list(V=diag(2), nu=0.02, alpha.mu=c(0,0),alpha.V=diag(2)*1000), G2=list(V=diag(2), nu=0.02, alpha.mu=c(0,0),alpha.V=diag(2)*1000), G3=list(V=diag(2), nu=0.02, alpha.mu=c(0,0),alpha.V=diag(2)*1000)))**

**model3 <- MCMCglmm(AGG~SEX, random=~us(SEX):animal + us(SEX):PE + us(SEX):Idf, rcov=~idh(SEX):units, prior=prior2.6, pedigree=AggPed, data=Data4A, nitt = 5000000, thin = 100, burnin = 150000)**

Summary

Iterations = 150001:4999901

Thinning interval = 100

Sample size = 48500

DIC: 1612.446

G-structure: ~us(SEX):animal

post.mean l-95% CI u-95% CI eff.samp

SEX1:SEX1.animal 2.113 1.786e-09 5.399 33025 ***#* female *V_A_***

SEX2:SEX1.animal 5.576 -1.117e+01 20.839 21602 **# female:male *COV_A_***

SEX1:SEX2.animal 5.576 -1.117e+01 20.839 21602

SEX2:SEX2.animal 77.084 2.451e+01 134.729 46918 ***#* male *V_A_***

~us(SEX):PE

post.mean l-95% CI u-95% CI eff.samp

SEX1:SEX1.PE 3.936 1.943e-09 11.335 46488 ***#* female *V_CE/M_***

SEX2:SEX1.PE -1.361 -1.349e+01 8.059 47724

SEX1:SEX2.PE -1.361 -1.349e+01 8.059 47724

SEX2:SEX2.PE 12.082 1.477e-10 47.063 48500 ***#* male *V_CE/M_***

~us(SEX):Idf

post.mean l-95% CI u-95% CI eff.samp

SEX1:SEX1.Idf 11.724 6.028 17.98 32939 ***#* female *V_C_***

SEX2:SEX1.Idf 5.034 -29.701 37.20 17626

SEX1:SEX2.Idf 5.034 -29.701 37.20 17626

SEX2:SEX2.Idf 113.237 44.966 185.49 47387 ***#* male *V_C_***

R-structure: ~idh(SEX):units

post.mean l-95% CI u-95% CI eff.samp

SEX1.units 3.327 1.476 5.537 45659 ***#* female *V_R_***

SEX2.units 62.783 28.339 103.386 45898 ***#* male *V_R_***

Location effects: AGG ~ SEX

post.mean l-95% CI u-95% CI eff.samp pMCMC

(Intercept) 2.136 1.195 3.035 45045 <2e-05 ***

SEX2 11.340 7.457 15.292 48500 <2e-05 *** **# fixed effect**

**---**

Signif. codes: 0 ‘***’ 0.001 ‘**’ 0.01 ‘*’ 0.05 ‘.’ 0.1 ‘ ’ 1

> posterior.mode(model3$Sol[, "SEX2"])

var1

11.20768

> plot(model3$Sol)

> plot(model3$VCV)

> effectiveSize(model3$VCV)

SEX1:SEX1.animal SEX2:SEX1.animal SEX1:SEX2.animal SEX2:SEX2.animal SEX1:SEX1.PE SEX2:SEX1.PE SEX1:SEX2.PE SEX2:SEX2.PE

33024.81 21601.52 21601.52 46917.55 46487.54 47723.59 47723.59 48500.00

SEX1:SEX1.Idf SEX2:SEX1.Idf SEX1:SEX2.Idf SEX2:SEX2.Idf SEX1.units SEX2.units

32939.17 17626.06 17626.06 47386.54 45659.22 45898.35

> heidel.diag(model3$VCV)

Stationarity start p-value

test iteration

SEX1:SEX1.animal passed 1 0.757

SEX2:SEX1.animal passed 1 0.438

SEX1:SEX2.animal passed 1 0.438

SEX2:SEX2.animal passed 1 0.827

SEX1:SEX1.PE passed 1 0.225

SEX2:SEX1.PE passed 1 0.741

SEX1:SEX2.PE passed 1 0.741

SEX2:SEX2.PE passed 1 0.063

SEX1:SEX1.Idf passed 1 0.912

SEX2:SEX1.Idf passed 1 0.129

SEX1:SEX2.Idf passed 1 0.129

SEX2:SEX2.Idf passed 1 0.588

SEX1.units passed 1 0.842

SEX2.units passed 1 0.672

Halfwidth Mean Halfwidth

test

SEX1:SEX1.animal passed 2.11 0.0229

SEX2:SEX1.animal passed 5.58 0.1084

SEX1:SEX2.animal passed 5.58 0.1084

SEX2:SEX2.animal passed 77.08 0.2582

SEX1:SEX1.PE passed 3.94 0.0355

SEX2:SEX1.PE passed -1.36 0.0467

SEX1:SEX2.PE passed -1.36 0.0467

SEX2:SEX2.PE passed 12.08 0.1639

SEX1:SEX1.Idf passed 11.72 0.0327

SEX2:SEX1.Idf passed 5.03 0.2657

SEX1:SEX2.Idf passed 5.03 0.2657

SEX2:SEX2.Idf passed 113.24 0.3250

SEX1.units passed 3.33 0.0103

SEX2.units passed 62.78 0.1869

> HPDinterval(model3$VCV)

lower upper

SEX1:SEX1.animal 1.786071e-09 5.398968

SEX2:SEX1.animal -1.117123e+01 20.838623

SEX1:SEX2.animal -1.117123e+01 20.838623

SEX2:SEX2.animal 2.450947e+01 134.729107

SEX1:SEX1.PE 1.942834e-09 11.334858

SEX2:SEX1.PE -1.348822e+01 8.059113

SEX1:SEX2.PE -1.348822e+01 8.059113

SEX2:SEX2.PE 1.477017e-10 47.062915

SEX1:SEX1.Idf 6.027860e+00 17.976510

SEX2:SEX1.Idf -2.970092e+01 37.204263

SEX1:SEX2.Idf -2.970092e+01 37.204263

SEX2:SEX2.Idf 4.496616e+01 185.490576

SEX1.units 1.475656e+00 5.537012

SEX2.units 2.833931e+01 103.386026

attr(,"Probability")

[1] 0.95

> vAf3 <- model3$VCV[,'SEX1:SEX1.animal']

> vRf3 <- model3$VCV[,'SEX1.units']

> VPEf3<- model3$VCV[,'SEX1:SEX1.PE']

> vIdff3 <- model3$VCV[,'SEX1:SEX1.Idf']

>

>

> vAm3 <- model3$VCV[,'SEX2:SEX2.animal']

> vRm3 <- model3$VCV[,'SEX2.units']

> VPEm3<- model3$VCV[,'SEX2:SEX2.PE']

> vIdfm3 <- model3$VCV[,'SEX2:SEX2.Idf']

>

>

> mean(vAf3)

[1]

> HPDinterval(vAf3)

lower upper

var1 1.786071e-09 5.398968

attr(,"Probability")

[1] 0.95

>

> mean(vRf3)

[1] 3.327242

> HPDinterval(vRf3)

lower upper

var1 1.475656 5.537012

attr(,"Probability")

[1] 0.95

>

> mean(VPEf3)

[1] 3.936001

> HPDinterval(VPEf3)

lower upper

var1 1.942834e-09 11.33486

attr(,"Probability")

[1] 0.95

>

> mean(vIdff3)

[1] 11.72354

> HPDinterval(vIdff3)

lower upper

var1 6.02786 17.97651

attr(,"Probability")

[1] 0.95

>

>

> mean(vAm3)

[1] 77.08375

> HPDinterval(vAm3)

lower upper

var1 24.50947 134.7291

attr(,"Probability")

[1] 0.95

>

> mean(vRm3)

[1] 62.78307

> HPDinterval(vRm3)

lower upper

var1 28.33931 103.386

attr(,"Probability")

[1] 0.95

>

> mean(VPEm3)

[1] 12.08206

> HPDinterval(VPEm3)

lower upper

var1 1.477017e-10 47.06292

attr(,"Probability")

[1] 0.95

>

> mean(vIdfm3)

[1] 113.2368

> HPDinterval(vIdfm3)

lower upper

var1 44.96616 185.4906

attr(,"Probability")

[1] 0.95

>

> vf3 <- sapply(1:nrow(model3[["Sol"]]), function(i) {var(predict(model3, it = i)) })

>

> mean(vf3)

[1] 32.98287

>

> heritF3.2<- vAf3/(vAf3 + vRf3 + VPEf3 + vIdff3 + vf3) **# female heritability:** $\boldsymbol{h}^{\boldsymbol{2}}\mathbf{=}\frac{\boldsymbol{V}_{\boldsymbol{A}}}{\left( \boldsymbol{V}_{\boldsymbol{A}}\mathbf{+}{\boldsymbol{V}_{\boldsymbol{CE/M}}\boldsymbol{+ V}}_{\boldsymbol{R}}\mathbf{+}\boldsymbol{V}_{\boldsymbol{C}}\boldsymbol{+}\boldsymbol{V}_{\boldsymbol{f}} \right)}$

> mean(heritF3.2)

[1] 0.04044751

> HPDinterval(heritF3.2)

lower upper

var1 2.886888e-11 0.1068541

attr(,"Probability")

[1] 0.95

>

> heritM3.2<- vAm3/(vAm3 + vRm3 + VPEm3+ vIdfm3 + vf3) **# male heritability:** $\boldsymbol{h}^{\boldsymbol{2}}\mathbf{=}\frac{\boldsymbol{V}_{\boldsymbol{A}}}{\left( \boldsymbol{V}_{\boldsymbol{A}}\mathbf{+}{\boldsymbol{V}_{\boldsymbol{CE/M}}\boldsymbol{+ V}}_{\boldsymbol{R}}\mathbf{+}\boldsymbol{V}_{\boldsymbol{C}}\boldsymbol{+}\boldsymbol{V}_{\boldsymbol{f}} \right)}$

> mean(heritM3.2)

[1] 0.2588037

> HPDinterval(heritM3.2)

lower upper

var1 0.09115065 0.4282608

attr(,"Probability")

[1] 0.95

>

>

> herit.diff3.2<- heritF3.2 - heritM3.2 # **sex difference in heritability (female heritability – male heritability)**

> mean(herit.diff3.2)

[1] -0.2183562

> HPDinterval(herit.diff3.2)

lower upper

var1 -0.4033486 -0.03496377

attr(,"Probability")

[1] 0.95

>

>

> corr.gen1 <- model3$VCV[, 'SEX1:SEX2.animal']/sqrt((model3$VCV[,'SEX1:SEX1.animal']*model3$VCV[,'SEX2:SEX2.animal'])) **# cross-sex gemetic correlation *r_mf_***

>

> mean(corr.gen1)

[1] 0.4547135

>

> HPDinterval(corr.gen1)

lower upper

var1 -0.9109348 1

attr(,"Probability")

[1] 0.95

>

>

>

>

>

> CVAf3<- sqrt(model3$VCV[,'SEX1:SEX1.animal'])/2.2 ***#* female *CV_A_***

> mean(CVAf3)

[1] 0.5947628

> HPDinterval(CVAf3)

lower upper

var1 1.920997e-05 1.056167

attr(,"Probability")

[1] 0.95

>

> If3<- model3$VCV[,'SEX1:SEX1.animal']/4.84 ***#* female *I***

> mean(If3)

[1] 0.4366128

> HPDinterval(If3)

lower upper

var1 3.690229e-10 1.115489

attr(,"Probability")

[1] 0.95

>

> CVRf3<- sqrt(model3$VCV[,'SEX1.units'])/2.2 ***#* female *CV_R_***

> mean(CVRf3)

[1] 0.8181004

> HPDinterval(CVRf3)

lower upper

var1 0.5767143 1.089752

attr(,"Probability")

[1] 0.95

>

>

> CVPEf3<- sqrt(model3$VCV[,'SEX1:SEX1.PE'])/2.2 ***#* female *CV_CE/M_***

> mean(CVPEf3)

[1] 0.7925874

> HPDinterval(CVPEf3)

lower upper

var1 2.003527e-05 1.530331

attr(,"Probability")

[1] 0.95

>

> CVAm3<- sqrt(model3$VCV[,'SEX2:SEX2.animal'])/13.143 ***#* male *CV_A_***

> mean(CVAm3)

[1] 0.6561927

> HPDinterval(CVAm3)

lower upper

var1 0.4175771 0.9124269

attr(,"Probability")

[1] 0.95

>

> Im3<- model3$VCV[,'SEX2:SEX2.animal']/172.738 ***#* male *I***

> mean(Im3)

[1] 0.4462466

> HPDinterval(Im3)

lower upper

var1 0.1418881 0.7799622

attr(,"Probability")

[1] 0.95

>

> CVRm3<- sqrt(model3$VCV[,'SEX2.units'])/13.143 ***#* male *CV_R_***

> mean(CVRm3)

[1] 0.5953911

> HPDinterval(CVRm3)

lower upper

var1 0.4154631 0.7809528

attr(,"Probability")

[1] 0.95

>

> CVPEm3<- sqrt(model3$VCV[,'SEX2:SEX2.PE'])/13.143 ***#* male *CV_CE/M_***

> mean(CVPEm3)

[1] 0.2087011

> HPDinterval(CVPEm3)

lower upper

var1 9.246944e-07 0.5219692

attr(,"Probability")

[1] 0.95

>

>

> CVA.diff3 <- CVAf3 - CVAm3 # **sex difference in *CV_A_***

> mean(CVA.diff3)

[1] -0.06142996

> HPDinterval(CVA.diff3)

lower upper

var1 -0.6943243 0.5066605

attr(,"Probability")

[1] 0.95

>

> I.diff3 <- If3 - Im3 # **sex difference in *I***

> mean(I.diff3)

[1] -0.009633849

> HPDinterval(I.diff3)

lower upper

var1 -0.7269988 0.7864392

attr(,"Probability")

[1] 0.95

>

> CVR.diff3 <- CVRf3 - CVRm3 # **sex difference in *CV_R_***

> mean(CVR.diff3)

[1] 0.2227092

> HPDinterval(CVR.diff3)

lower upper

var1 -0.09245532 0.5493336

attr(,"Probability")

[1] 0.95

>

>

> CVPE.diff3<- CVPEf3 - CVPEm3 # **sex difference in *CV_CE/M_***

> mean(CVPE.diff3)

[1] 0.5838863

> HPDinterval(CVPE.diff3)

lower upper

var1 -0.2872713 1.467156

attr(,"Probability")

[1] 0.95

## Prior 2

**prior2.4 <- list(R=list(V=diag(2), nu=2), G=list(G1=list(V=diag(2), nu=0.002, alpha.mu=c(0,0),alpha.V=diag(2)*1000), G2=list(V=diag(2), nu=0.002, alpha.mu=c(0,0),alpha.V=diag(2)*1000), G3=list(V=diag(2), nu=0.002, alpha.mu=c(0,0),alpha.V=diag(2)*1000)))**

**model3 <- MCMCglmm(AGG~SEX, random=~us(SEX):animal + us(SEX):PE + us(SEX):Idf, rcov=~idh(SEX):units, prior=prior2.4, pedigree=AggPed, data=Data4A, nitt=15000000, burnin=150000, thin=100)**

Summary

Iterations = 150001:14999901

Thinning interval = 100

Sample size = 148500

DIC: 1616.968

G-structure: ~us(SEX):animal

post.mean l-95% CI u-95% CI eff.samp

SEX1:SEX1.animal 2.139 2.903e-10 5.599 93868

SEX2:SEX1.animal 6.985 -1.199e+01 23.117 56724

SEX1:SEX2.animal 6.985 -1.199e+01 23.117 56724

SEX2:SEX2.animal 78.255 2.471e+01 138.741 115141

~us(SEX):PE

post.mean l-95% CI u-95% CI eff.samp

SEX1:SEX1.PE 3.907 6.760e-09 11.22 117544

SEX2:SEX1.PE -1.647 -1.379e+01 8.02 133502

SEX1:SEX2.PE -1.647 -1.379e+01 8.02 133502

SEX2:SEX2.PE 10.000 1.348e-09 40.87 143935

~us(SEX):Idf

post.mean l-95% CI u-95% CI eff.samp

SEX1:SEX1.Idf 11.520 5.765 18.05 98819

SEX2:SEX1.Idf 7.331 -32.020 40.81 31539

SEX1:SEX2.Idf 7.331 -32.020 40.81 31539

SEX2:SEX2.Idf 110.648 41.332 184.45 123276

R-structure: ~idh(SEX):units

post.mean l-95% CI u-95% CI eff.samp

SEX1.units 3.405 1.50 5.722 134254

SEX2.units 64.627 28.54 107.697 115437

Location effects: AGG ~ SEX

post.mean l-95% CI u-95% CI eff.samp pMCMC

(Intercept) 2.131 1.213 3.049 145403 1.35e-05 ***

SEX2 11.372 7.516 15.255 148500 < 7e-06 ***

---

Signif. codes: 0 ‘***’ 0.001 ‘**’ 0.01 ‘*’ 0.05 ‘.’ 0.1 ‘ ’ 1

> posterior.mode(model3$Sol[, "SEX2"])

var1

11.49329

> plot(model3$Sol)

> plot(model3$VCV)

> effectiveSize(model3$VCV)

SEX1:SEX1.animal SEX2:SEX1.animal SEX1:SEX2.animal SEX2:SEX2.animal SEX1:SEX1.PE SEX2:SEX1.PE SEX1:SEX2.PE

93868.05 56723.52 56723.52 115141.43 117543.98 133502.46 133502.46

SEX2:SEX2.PE SEX1:SEX1.Idf SEX2:SEX1.Idf SEX1:SEX2.Idf SEX2:SEX2.Idf SEX1.units SEX2.units

143934.79 98818.96 31539.45 31539.45 123276.27 134254.14 115436.63

> heidel.diag(model3$VCV)

Stationarity start p-value

test iteration

SEX1:SEX1.animal passed 1 0.8319

SEX2:SEX1.animal passed 1 0.9110

SEX1:SEX2.animal passed 1 0.9110

SEX2:SEX2.animal passed 1 0.2358

SEX1:SEX1.PE passed 1 0.0523

SEX2:SEX1.PE passed 1 0.2789

SEX1:SEX2.PE passed 1 0.2789

SEX2:SEX2.PE passed 1 0.2129

SEX1:SEX1.Idf passed 1 0.7347

SEX2:SEX1.Idf passed 1 0.4706

SEX1:SEX2.Idf passed 1 0.4706

SEX2:SEX2.Idf passed 1 0.0551

SEX1.units passed 1 0.8262

SEX2.units passed 1 0.2414

Halfwidth Mean Halfwidth

test

SEX1:SEX1.animal passed 2.14 0.01430

SEX2:SEX1.animal passed 6.98 0.07197

SEX1:SEX2.animal passed 6.98 0.07197

SEX2:SEX2.animal passed 78.25 0.17054

SEX1:SEX1.PE passed 3.91 0.02191

SEX2:SEX1.PE passed -1.65 0.02846

SEX1:SEX2.PE passed -1.65 0.02846

SEX2:SEX2.PE passed 10.00 0.08646

SEX1:SEX1.Idf passed 11.52 0.01924

SEX2:SEX1.Idf passed 7.33 0.22389

SEX1:SEX2.Idf passed 7.33 0.22389

SEX2:SEX2.Idf passed 110.65 0.20416

SEX1.units passed 3.40 0.00627

SEX2.units passed 64.63 0.12475

> HPDinterval(model3$VCV)

lower upper

SEX1:SEX1.animal 2.903481e-10 5.599279

SEX2:SEX1.animal -1.198507e+01 23.116504

SEX1:SEX2.animal -1.198507e+01 23.116504

SEX2:SEX2.animal 2.471285e+01 138.740909

SEX1:SEX1.PE 6.760405e-09 11.217546

SEX2:SEX1.PE -1.379290e+01 8.020250

SEX1:SEX2.PE -1.379290e+01 8.020250

SEX2:SEX2.PE 1.347718e-09 40.868854

SEX1:SEX1.Idf 5.764550e+00 18.047003

SEX2:SEX1.Idf -3.201973e+01 40.810443

SEX1:SEX2.Idf -3.201973e+01 40.810443

SEX2:SEX2.Idf 4.133249e+01 184.448918

SEX1.units 1.499642e+00 5.721532

SEX2.units 2.854225e+01 107.696849

attr(,"Probability")

[1] 0.95

> vAf3 <- model3$VCV[,'SEX1:SEX1.animal']

> vRf3 <- model3$VCV[,'SEX1.units']

> VPEf3<- model3$VCV[,'SEX1:SEX1.PE']

> vIdff3 <- model3$VCV[,'SEX1:SEX1.Idf']

>

>

> vAm3 <- model3$VCV[,'SEX2:SEX2.animal']

> vRm3 <- model3$VCV[,'SEX2.units']

> VPEm3<- model3$VCV[,'SEX2:SEX2.PE']

> vIdfm3 <- model3$VCV[,'SEX2:SEX2.Idf']

>

>

> mean(vAf3)

[1] 2.138515

> HPDinterval(vAf3)

lower upper

var1 2.903481e-10 5.599279

attr(,"Probability")

[1] 0.95

>

> mean(vRf3)

[1] 3.404939

> HPDinterval(vRf3)

lower upper

var1 1.499642 5.721532

attr(,"Probability")

[1] 0.95

>

> mean(VPEf3)

[1] 3.90686

> HPDinterval(VPEf3)

lower upper

var1 6.760405e-09 11.21755

attr(,"Probability")

[1] 0.95

>

> mean(vIdff3)

[1] 11.51951

> HPDinterval(vIdff3)

lower upper

var1 5.76455 18.047

attr(,"Probability")

[1] 0.95

>

>

> mean(vAm3)

[1] 78.2549

> HPDinterval(vAm3)

lower upper

var1 24.71285 138.7409

attr(,"Probability")

[1] 0.95

>

> mean(vRm3)

[1] 64.62703

> HPDinterval(vRm3)

lower upper

var1 28.54225 107.6968

attr(,"Probability")

[1] 0.95

>

> mean(VPEm3)

[1] 10.00031

> HPDinterval(VPEm3)

lower upper

var1 1.347718e-09 40.86885

attr(,"Probability")

[1] 0.95

>

> mean(vIdfm3)

[1] 110.648

> HPDinterval(vIdfm3)

lower upper

var1 41.33249 184.4489

attr(,"Probability")

[1] 0.95

>

> vf3 <- sapply(1:nrow(model3[["Sol"]]), function(i) {var(predict(model3, it = i)) })

>

> mean(vf3)

[1] 33.14203

>

>

> heritF3.2<- vAf3/(vAf3 + vRf3 + VPEf3 + vIdff3 + vf3)

> mean(heritF3.2)

[1] 0.04071437

> HPDinterval(heritF3.2)

lower upper

var1 5.165879e-12 0.1097536

attr(,"Probability")

[1] 0.95

>

> heritM3.2<- vAm3/(vAm3 + vRm3 + VPEm3+ vIdfm3 + vf3)

> mean(heritM3.2)

[1] 0.2636849

> HPDinterval(heritM3.2)

lower upper

var1 0.09413866 0.4413482

attr(,"Probability")

[1] 0.95

>

> herit.diff3.2<- heritF3.2 - heritM3.2

> mean(herit.diff3.2)

[1] -0.2229705

> HPDinterval(herit.diff3.2)

lower upper

var1 -0.4167724 -0.03827381

attr(,"Probability")

[1] 0.95

>

>

> corr.gen1 <- model3$VCV[, 'SEX1:SEX2.animal']/sqrt((model3$VCV[,'SEX1:SEX1.animal']*model3$VCV[,'SEX2:SEX2.animal']))

>

> mean(corr.gen1)

[1] 0.563133

>

> HPDinterval(corr.gen1)

lower upper

var1 -0.9683882 1

attr(,"Probability")

[1] 0.95

> CVAf3<- sqrt(model3$VCV[,'SEX1:SEX1.animal'])/2.2

> mean(CVAf3)

[1] 0.5948226

> HPDinterval(CVAf3)

lower upper

var1 7.745274e-06 1.075582

attr(,"Probability")

[1] 0.95

>

> If3<- model3$VCV[,'SEX1:SEX1.animal']/4.84

> mean(If3)

[1] 0.441842

> HPDinterval(If3)

lower upper

var1 5.998927e-11 1.156876

attr(,"Probability")

[1] 0.95

>

> CVRf3<- sqrt(model3$VCV[,'SEX1.units'])/2.2

> mean(CVRf3)

[1] 0.8272237

> HPDinterval(CVRf3)

lower upper

var1 0.5778622 1.102224

attr(,"Probability")

[1] 0.95

>

>

> CVPEf3<- sqrt(model3$VCV[,'SEX1:SEX1.PE'])/2.2

> mean(CVPEf3)

[1] 0.7903251

> HPDinterval(CVPEf3)

lower upper

var1 0.0001040445 1.522457

attr(,"Probability")

[1] 0.95

>

> CVAm3<- sqrt(model3$VCV[,'SEX2:SEX2.animal'])/13.143

> mean(CVAm3)

[1] 0.6606286

> HPDinterval(CVAm3)

lower upper

var1 0.4130273 0.9206825

attr(,"Probability")

[1] 0.95

>

> Im3<- model3$VCV[,'SEX2:SEX2.animal']/172.738

> mean(Im3)

[1] 0.4530266

> HPDinterval(Im3)

lower upper

var1 0.1430655 0.803187

attr(,"Probability")

[1] 0.95

>

> CVRm3<- sqrt(model3$VCV[,'SEX2.units'])/13.143

> mean(CVRm3)

[1] 0.6036831

> HPDinterval(CVRm3)

lower upper

var1 0.4201712 0.7996901

attr(,"Probability")

[1] 0.95

>

> CVPEm3<- sqrt(model3$VCV[,'SEX2:SEX2.PE'])/13.143

> mean(CVPEm3)

[1] 0.184892

> HPDinterval(CVPEm3)

lower upper

var1 2.793219e-06 0.4864091

attr(,"Probability")

[1] 0.95

>

>

> CVA.diff3 <- CVAf3 - CVAm3

> mean(CVA.diff3)

[1] -0.06580602

> HPDinterval(CVA.diff3)

lower upper

var1 -0.693451 0.5311186

attr(,"Probability")

[1] 0.95

>

> I.diff3 <- If3 - Im3

> mean(I.diff3)

[1] -0.01118459

> HPDinterval(I.diff3)

lower upper

var1 -0.73872 0.8352568

attr(,"Probability")

[1] 0.95

>

> CVR.diff3 <- CVRf3 - CVRm3

> mean(CVR.diff3)

[1] 0.2235405

> HPDinterval(CVR.diff3)

lower upper

var1 -0.1060461 0.5620081

attr(,"Probability")

[1] 0.95

>

>

> CVPE.diff3<- CVPEf3 - CVPEm3

> mean(CVPE.diff3)

[1] 0.6054331

> HPDinterval(CVPE.diff3)

lower upper

var1 -0.2304294 1.485357

attr(,"Probability")

[1] 0.95

### **­­­­­­­­­­­­­­­­­­­­**

# ACTIVITY

## ## Model 1 (fixed factor = none, random = animal + common environment/maternal effect)

### Prior 1

**prior1<-list(G = list(G1 = list(V = 1, nu = 0.002), G2 = list(V = 1, nu = 0.002)), R = list(V = 1, nu = 0.002))**

**model4 <- MCMCglmm(NS ~ 1, random = ~animal + PE, pedigree = NA_ped,data = Data5, nitt = 5000000, thin = 100, burnin = 150000, prior = prior1, verbose = FALSE)**

Summary

Iterations = 150001:4999901

Thinning interval = 100

Sample size = 48500

DIC: 356.1541

G-structure: ~animal

post.mean l-95% CI u-95% CI eff.samp

animal 0.1268 0.06165 0.1976 49552

~PE

post.mean l-95% CI u-95% CI eff.samp

PE 0.3156 0.09457 0.5989 48500

R-structure: ~units

post.mean l-95% CI u-95% CI eff.samp

units 0.1433 0.1025 0.1873 48500

Location effects: NS ~ 1

post.mean l-95% CI u-95% CI eff.samp pMCMC

(Intercept) 0.7172 0.6261 0.8073 48500 <2e-05 ***

---

Signif. codes: 0 ‘***’ 0.001 ‘**’ 0.01 ‘*’ 0.05 ‘.’ 0.1 ‘ ’ 1

> plot(model4$Sol)

> plot(model4$VCV)

> autocorr.diag(model4$Sol)

(Intercept)

Lag 0 1.000000000

Lag 100 0.001117850

Lag 500 0.002415283

Lag 1000 0.001130710

Lag 5000 0.001463298

> autocorr.diag(model4$VCV)

animal PE units

Lag 0 1.000000000 1.000000000 1.0000000000

Lag 100 -0.003375917 0.001304638 -0.0011065363

Lag 500 -0.006200511 0.003083017 0.0095871318

Lag 1000 -0.001170720 0.001493104 -0.0033629591

Lag 5000 -0.004874206 -0.004391754 -0.0006123928

> effectiveSize(model4$Sol)

(Intercept)

48500

> effectiveSize(model4$VCV)

animal PE units

49552.14 48500.00 48500.00

> heidel.diag(model4$VCV)

Stationarity start p-value

test iteration

animal passed 1 0.178

PE passed 1 0.384

units passed 1 0.320

Halfwidth Mean Halfwidth

test

animal passed 0.127 0.000307

PE passed 0.316 0.001292

units passed 0.143 0.000197

> HPDinterval(model4$VCV)

lower upper

animal 0.0616536 0.1975598

PE 0.0945655 0.5988801

units 0.1024710 0.1872797

attr(,"Probability")

[1] 0.95

> vA4 <- model4$VCV[,'animal']

> vR4 <- model4$VCV[,'units']

> vPE4 <- model4$VCV[,'PE']

>

> mean(vA4)

[1] 0.1267788

> HPDinterval(vA4)

lower upper

var1 0.0616536 0.1975598

attr(,"Probability")

[1] 0.95

>

> mean(vR4)

[1] 0.1432823

> HPDinterval(vR4)

lower upper

var1 0.102471 0.1872797

attr(,"Probability")

[1] 0.95

>

>

> mean(vPE4)

[1] 0.3156439

> HPDinterval(vPE4)

lower upper

var1 0.0945655 0.5988801

attr(,"Probability")

[1] 0.95

>

>

> herit4<- vA4/(vA4 + vR4 + vPE4)

> mean(herit4)

[1] 0.2246433

> HPDinterval(herit4)

lower upper

var1 0.09458001 0.3578916

attr(,"Probability")

[1] 0.95

>

> CVA4<- sqrt(model4$VCV[, "animal"])/3.62

>

> mean(CVA4)

[1] 0.09739756

> HPDinterval(CVA4)

lower upper

var1 0.07031342 0.1242105

attr(,"Probability")

[1] 0.95

>

> I4<- model4$VCV[, "animal"]/13.071

> mean(I4)

[1] 0.009699241

> HPDinterval(I4)

lower upper

var1 0.004716823 0.01511436

attr(,"Probability")

[1] 0.95

>

> CVR4<- sqrt(model4$VCV[, "units"])/3.62

> mean(CVR4)

[1] 0.1042596

> HPDinterval(CVR4)

lower upper

var1 0.08935326 0.1203847

attr(,"Probability")

[1] 0.95

>

>

> CVPE4<- sqrt(model4$VCV[, "PE"])/3.62

> mean(CVPE4)

[1] 0.1516026

> HPDinterval(CVPE4)

lower upper

var1 0.09074812 0.2170668

attr(,"Probability")

[1] 0.95

_____________________________________________________________________

### Prior 2

**prior1.1<-list(G=list(G1=list(V=matrix(p.var*0.5),n=1), G2 = list(V = 1, nu = 0.002)),R=list(V=matrix(p.var*0.5),n=1))**

**model4 <- MCMCglmm(NS ~ 1, random = ~animal + PE, pedigree = NA_ped,data = Data5, nitt = 5000000, thin = 100, burnin = 150000, prior = prior1.1, verbose = FALSE)**

Summary

Iterations = 150001:4999901

Thinning interval = 100

Sample size = 48500

DIC: 355.4046

G-structure: ~animal

post.mean l-95% CI u-95% CI eff.samp

animal 0.1284 0.06485 0.1954 50023

~PE

post.mean l-95% CI u-95% CI eff.samp

PE 0.3168 0.09482 0.5982 48500

R-structure: ~units

post.mean l-95% CI u-95% CI eff.samp

units 0.1426 0.1029 0.1845 49418

Location effects: NS ~ 1

post.mean l-95% CI u-95% CI eff.samp pMCMC

(Intercept) 0.7170 0.6294 0.8094 48500 <2e-05 ***

---

Signif. codes: 0 ‘***’ 0.001 ‘**’ 0.01 ‘*’ 0.05 ‘.’ 0.1 ‘ ’ 1

> plot(model4$Sol)

> plot(model4$VCV)

> autocorr.diag(model4$Sol)

(Intercept)

Lag 0 1.0000000000

Lag 100 0.0056112817

Lag 500 0.0001803159

Lag 1000 0.0077985867

Lag 5000 0.0020962495

> autocorr.diag(model4$VCV)

animal PE units

Lag 0 1.000000000 1.000000000 1.000000000

Lag 100 -0.003327344 0.002511403 -0.009380548

Lag 500 -0.006304752 0.005192560 0.005949557

Lag 1000 -0.006397587 -0.009030776 -0.003838128

Lag 5000 0.006918498 0.001972209 -0.009059943

> effectiveSize(model4$Sol)

(Intercept)

48500

> effectiveSize(model4$VCV)

animal PE units

50023.07 48500.00 49417.51

> heidel.diag(model4$VCV)

Stationarity start p-value

test iteration

animal passed 1 0.807

PE passed 1 0.702

units passed 1 0.853

Halfwidth Mean Halfwidth

test

animal passed 0.128 0.000293

PE passed 0.317 0.001296

units passed 0.143 0.000187

> HPDinterval(model4$VCV)

lower upper

animal 0.06485007 0.1954163

PE 0.09482014 0.5981754

units 0.10289993 0.1845008

attr(,"Probability")

[1] 0.95

> vA4 <- model4$VCV[,'animal']

> vR4 <- model4$VCV[,'units']

> vPE4 <- model4$VCV[,'PE']

>

> mean(vA4)

[1] 0.1283735

> HPDinterval(vA4)

lower upper

var1 0.06485007 0.1954163

attr(,"Probability")

[1] 0.95

>

> mean(vR4)

[1] 0.1426091

> HPDinterval(vR4)

lower upper

var1 0.1028999 0.1845008

attr(,"Probability")

[1] 0.95

>

>

> mean(vPE4)

[1] 0.3168228

> HPDinterval(vPE4)

lower upper

var1 0.09482014 0.5981754

attr(,"Probability")

[1] 0.95

>

>

> herit4<- vA4/(vA4 + vR4 + vPE4)

> mean(herit4)

[1] 0.2268383

> HPDinterval(herit4)

lower upper

var1 0.1002932 0.356514

attr(,"Probability")

[1] 0.95

>

> CVA4<- sqrt(model4$VCV[, "animal"])/3.62

>

> mean(CVA4)

[1] 0.09811701

> HPDinterval(CVA4)

lower upper

var1 0.07267487 0.1239442

attr(,"Probability")

[1] 0.95

>

> I4<- model4$VCV[, "animal"]/13.071

> mean(I4)

[1] 0.009821243

> HPDinterval(I4)

lower upper

var1 0.004961371 0.01495037

attr(,"Probability")

[1] 0.95

>

> CVR4<- sqrt(model4$VCV[, "units"])/3.62

> mean(CVR4)

[1] 0.1040369

> HPDinterval(CVR4)

lower upper

var1 0.08953285 0.1194124

attr(,"Probability")

[1] 0.95

>

>

> CVPE4<- sqrt(model4$VCV[, "PE"])/3.62

> mean(CVPE4)

[1] 0.1519044

> HPDinterval(CVPE4)

lower upper

var1 0.09201499 0.2177562

attr(,"Probability")

[1] 0.95

XXXXXXXXXXXXXXXXXXXXXXXXXXXXXXXXXXXXXXXXXXXXXXXXXXXXXXXXX

## ## Model 2 (fixed factor = sex, random = animal + common environment/maternal effect)

### Prior 1

**prior1<-list(G = list(G1 = list(V = 1, nu = 0.002), G2 = list(V = 1, nu = 0.002)), R = list(V = 1, nu = 0.002))**

**model5 <- MCMCglmm(NS ~ SEX, random = ~animal + PE, pedigree = NA_ped,data = Data5, nitt = 5000000, thin = 100, burnin = 150000, prior = prior1, verbose = FALSE)**

| Iterations = 150001:4999901  Thinning interval = 100  Sample size = 48500  DIC: 357.2538  G-structure: ~animal  post.mean l-95% CI u-95% CI eff.samp  animal 0.1233 0.05734 0.1923 48500  ~PE  post.mean l-95% CI u-95% CI eff.samp  PE 0.3153 0.09931 0.5992 48500  R-structure: ~units  post.mean l-95% CI u-95% CI eff.samp  units 0.1444 0.1037 0.1879 48500  Location effects: NS ~ SEX  post.mean l-95% CI u-95% CI eff.samp pMCMC  (Intercept) 0.67532 0.57347 0.78343 48500 <2e-05 ***  SEX2 0.09616 -0.03279 0.22397 48500 0.146  ---  Signif. codes: 0 ‘***’ 0.001 ‘**’ 0.01 ‘*’ 0.05 ‘.’ 0.1 ‘ ’ 1  > plot(model5$Sol)  > plot(model5$VCV)  > autocorr.diag(model5$Sol)  (Intercept) SEX2  Lag 0 1.000000000 1.000000000  Lag 100 0.003192466 0.002284887  Lag 500 -0.002006936 -0.004762417  Lag 1000 -0.004306336 -0.003796268  Lag 5000 0.002876621 -0.003557660  > autocorr.diag(model5$VCV)  animal PE units  Lag 0 1.0000000000 1.0000000000 1.0000000000  Lag 100 -0.0033860739 -0.0005696279 0.0026784170  Lag 500 -0.0001436577 0.0057905089 0.0003794170  Lag 1000 -0.0028298412 0.0010710023 0.0016118884  Lag 5000 0.0004597217 0.0062705430 -0.0008991004  > effectiveSize(model5$Sol)  (Intercept) SEX2  48500 48500  > effectiveSize(model5$VCV)  animal PE units  48500 48500 48500  > heidel.diag(model5$VCV)    Stationarity start p-value  test iteration  animal passed 1 0.950  PE passed 1 0.501  units passed 1 0.231    Halfwidth Mean Halfwidth  test  animal passed 0.123 0.000308  PE passed 0.315 0.001290  units passed 0.144 0.000196  > HPDinterval(model4$VCV)  lower upper  animal 0.0616536 0.1975598  PE 0.0945655 0.5988801  units 0.1024710 0.1872797  attr(,"Probability")  [1] 0.95  > vA5 <- model5$VCV[,'animal']  > vR5 <- model5$VCV[,'units']  > vPE5 <- model5$VCV[,'PE']  >  > mean(vA5)  [1] 0.1232583  > HPDinterval(vA5)  lower upper  var1 0.05734044 0.192296  attr(,"Probability")  [1] 0.95  >  > mean(vR5)  [1] 0.1444044  > HPDinterval(vR5)  lower upper  var1 0.1037203 0.1879474  attr(,"Probability")  [1] 0.95  >  > mean(vPE5)  [1] 0.3153485  > HPDinterval(vPE5)  lower upper  var1 0.09930555 0.5991651  attr(,"Probability")  [1] 0.95  >  > CVA5<- sqrt(model5$VCV[, "animal"])/3.62  > mean(CVA5)  [1] 0.09599013  > HPDinterval(CVA5)  lower upper  var1 0.06833398 0.1227165  attr(,"Probability")  [1] 0.95  >  > I5<- model5$VCV[, "animal"]/13.071  > mean(I5)  [1] 0.009429903  > HPDinterval(I5)  lower upper  var1 0.004386844 0.01471165  attr(,"Probability")  [1] 0.95  >  > CVR5<- sqrt(model5$VCV[, "units"])/3.62  > mean(CVR5)  [1] 0.1046734  > HPDinterval(CVR5)  lower upper  var1 0.08961392 0.1203012  attr(,"Probability")  [1] 0.95  >  > CVPE5<- sqrt(model5$VCV[, "PE"])/3.62  > mean(CVPE5)  [1] 0.1515595  > HPDinterval(CVPE5)  lower upper  var1 0.0933328 0.2183427  attr(,"Probability")  [1] 0.95  >  > Vf5 <- sapply(1:nrow(model5[["Sol"]]), function(i) {var(predict(model5, it = i)) }) |
| --- |
|  |
| > mean(Vf5)  [1] 0.003376631  > herit5.2<- vA5/(vA5 + vR5 + vPE5 + Vf5)  > mean(herit5.2)  [1] 0.2180204  > HPDinterval(herit5.2)  lower upper  var1 0.09240753 0.3501748  attr(,"Probability")  [1] 0.95 |

__________________________________________________________________________________

## Prior 2

**prior1.1<-list(G=list(G1=list(V=matrix(p.var*0.5),n=1), G2 = list(V = 1, nu = 0.002)),R=list(V=matrix(p.var*0.5),n=1))**

**model5 <- MCMCglmm(NS ~ SEX, random = ~animal + PE, pedigree = NA_ped,data = Data5, nitt = 5000000, thin = 100, burnin = 150000, prior = prior1.1, verbose = FALSE)**

Iterations = 150001:4999901

Thinning interval = 100

Sample size = 48500

DIC: 356.6265

G-structure: ~animal

post.mean l-95% CI u-95% CI eff.samp

animal 0.1248 0.06231 0.1924 48500

~PE

post.mean l-95% CI u-95% CI eff.samp

PE 0.3161 0.09514 0.5983 49137

R-structure: ~units

post.mean l-95% CI u-95% CI eff.samp

units 0.1435 0.1037 0.1866 48500

Location effects: NS ~ SEX

post.mean l-95% CI u-95% CI eff.samp pMCMC

(Intercept) 0.67505 0.57128 0.78160 48500 <2e-05 ***

SEX2 0.09641 -0.02662 0.22729 48500 0.139

---

Signif. codes: 0 ‘***’ 0.001 ‘**’ 0.01 ‘*’ 0.05 ‘.’ 0.1 ‘ ’ 1

> plot(model5$Sol)

> plot(model5$VCV)

> autocorr.diag(model5$Sol)

(Intercept) SEX2

Lag 0 1.000000000 1.000000000

Lag 100 0.002201617 0.003013779

Lag 500 0.007142964 -0.009438344

Lag 1000 -0.005077580 -0.001271148

Lag 5000 0.007462433 0.001457995

> autocorr.diag(model5$VCV)

animal PE units

Lag 0 1.0000000000 1.000000000 1.000000000

Lag 100 -0.0010081896 0.004400230 -0.002800020

Lag 500 0.0003405555 0.005336024 0.001147836

Lag 1000 0.0016696134 0.001193327 0.001648514

Lag 5000 0.0047925507 -0.004599693 -0.002115810

> effectiveSize(model5$Sol)

(Intercept) SEX2

48500 48500

> effectiveSize(model5$VCV)

animal PE units

48500.00 49137.41 48500.00

> heidel.diag(model5$VCV)

Stationarity start p-value

test iteration

animal passed 1 0.961

PE passed 9701 0.104

units passed 1 0.442

Halfwidth Mean Halfwidth

test

animal passed 0.125 0.000298

PE passed 0.317 0.001443

units passed 0.144 0.000191

> HPDinterval(model4$VCV)

lower upper

animal 0.06485007 0.1954163

PE 0.09482014 0.5981754

units 0.10289993 0.1845008

attr(,"Probability")

[1] 0.95

> vA5 <- model5$VCV[,'animal']

> vR5 <- model5$VCV[,'units']

> vPE5 <- model5$VCV[,'PE']

>

> mean(vA5)

[1] 0.1247937

> HPDinterval(vA5)

lower upper

var1 0.06230547 0.192377

attr(,"Probability")

[1] 0.95

>

> mean(vR5)

[1] 0.1435314

> HPDinterval(vR5)

lower upper

var1 0.1037044 0.1865786

attr(,"Probability")

[1] 0.95

>

> mean(vPE5)

[1] 0.3160601

> HPDinterval(vPE5)

lower upper

var1 0.09514053 0.5983093

attr(,"Probability")

[1] 0.95

>

> herit5<- vA5/(vA5 + vR5 + vPE5)

> mean(herit5)

[1] 0.2218246

> HPDinterval(herit5)

lower upper

var1 0.0984135 0.3535848

attr(,"Probability")

[1] 0.95

>

> CVA5<- sqrt(model5$VCV[, "animal"])/3.62

> mean(CVA5)

[1] 0.0966912

> HPDinterval(CVA5)

lower upper

var1 0.07112805 0.1227967

attr(,"Probability")

[1] 0.95

>

> I5<- model5$VCV[, "animal"]/13.071

> mean(I5)

[1] 0.009547371

> HPDinterval(I5)

lower upper

var1 0.004766695 0.01471785

attr(,"Probability")

[1] 0.95

>

> CVR5<- sqrt(model5$VCV[, "units"])/3.62

> mean(CVR5)

[1] 0.1043688

> HPDinterval(CVR5)

lower upper

var1 0.08950585 0.1197694

attr(,"Probability")

[1] 0.95

>

> CVPE5<- sqrt(model5$VCV[, "PE"])/3.62

> mean(CVPE5)

[1] 0.1517282

> HPDinterval(CVPE5)

lower upper

var1 0.09210876 0.2179492

attr(,"Probability")

[1] 0.95

>

> Vf5 <- sapply(1:nrow(model5[["Sol"]]), function(i) {var(predict(model5, it = i)) })

> mean(Vf5)

[1] 0.003367464

>

> herit5.2<- vA5/(vA5 + vR5 + vPE5 + Vf5)

> mean(herit5.2)

[1] 0.2204584

> HPDinterval(herit5.2)

lower upper

var1 0.09808682 0.3513532

attr(,"Probability")

[1] 0.95

XXXXXXXXXXXXXXXXXXXXXXXXXXXXXXXXXXXXXXXXXXXXXXXXXXXXXXXXX

## ## Model 3 (fixed factor = sex, random = us(SEX):animal + us(SEX):common environment/maternal effect)

### Prior 1

**prior2<-list(G = list(G1 = list(V=diag(2), nu = 0.002), G2 = list(V=diag(2), nu = 0.002)), R = list(V=diag(2), nu = 0.002))**

**model6 <- MCMCglmm(NS~SEX, random=~us(SEX):animal + us(SEX):PE, rcov=~idh(SEX):units, prior=prior2, pedigree=NA_ped, data=Data5, nitt=5000000, burnin=150000, thin=100)**

Summary

Iterations = 150001:4999901

Thinning interval = 100

Sample size = 48500

DIC: 361.1607

G-structure: ~us(SEX):animal

post.mean l-95% CI u-95% CI eff.samp

SEX1:SEX1.animal 0.10344 0.02117 0.1924 16226

SEX2:SEX1.animal 0.07389 -0.13259 0.1872 2399

SEX1:SEX2.animal 0.07389 -0.13259 0.1872 2399

SEX2:SEX2.animal 0.14673 0.04520 0.2513 21118

~us(SEX):PE

post.mean l-95% CI u-95% CI eff.samp

SEX1:SEX1.PE 0.2523 0.02421 0.5442 16794

SEX2:SEX1.PE 0.3060 0.07340 0.6102 25792

SEX1:SEX2.PE 0.3060 0.07340 0.6102 25792

SEX2:SEX2.PE 0.4185 0.09302 0.8585 46910

R-structure: ~idh(SEX):units

post.mean l-95% CI u-95% CI eff.samp

SEX1.units 0.1573 0.10078 0.2217 22165

SEX2.units 0.1372 0.08079 0.2021 28186

Location effects: NS ~ SEX

post.mean l-95% CI u-95% CI eff.samp pMCMC

(Intercept) 0.64831 0.52962 0.75950 23820 <2e-05 ***

SEX2 0.13641 -0.03333 0.31097 46564 0.121

---

Signif. codes: 0 ‘***’ 0.001 ‘**’ 0.01 ‘*’ 0.05 ‘.’ 0.1 ‘ ’ 1

> posterior.mode(model6$Sol[, "SEX2"])

var1

0.1322043

> plot(model6$Sol)

> plot(model6$VCV)

Hit <Return> to see next plot: autocorr.diag(model6$Sol)

Hit <Return> to see next plot: autocorr.diag(model6$VCV)

Hit <Return> to see next plot: effectiveSize(model6$Sol)

> effectiveSize(model6$VCV)

SEX1:SEX1.animal SEX2:SEX1.animal SEX1:SEX2.animal SEX2:SEX2.animal SEX1:SEX1.PE SEX2:SEX1.PE SEX1:SEX2.PE

16226.401 2398.583 2398.583 21118.101 16794.467 25791.746 25791.746

SEX2:SEX2.PE SEX1.units SEX2.units

46909.579 22164.558 28185.910

> heidel.diag(model6$VCV)

Stationarity start p-value

test iteration

SEX1:SEX1.animal passed 1 0.956

SEX2:SEX1.animal passed 1 0.972

SEX1:SEX2.animal passed 1 0.972

SEX2:SEX2.animal passed 1 0.385

SEX1:SEX1.PE passed 1 0.922

SEX2:SEX1.PE passed 1 0.656

SEX1:SEX2.PE passed 1 0.656

SEX2:SEX2.PE passed 1 0.376

SEX1.units passed 1 0.903

SEX2.units passed 1 0.572

Halfwidth Mean Halfwidth

test

SEX1:SEX1.animal passed 0.1034 0.000676

SEX2:SEX1.animal passed 0.0739 0.003387

SEX1:SEX2.animal passed 0.0739 0.003387

SEX2:SEX2.animal passed 0.1467 0.000713

SEX1:SEX1.PE passed 0.2523 0.002280

SEX2:SEX1.PE passed 0.3060 0.001885

SEX1:SEX2.PE passed 0.3060 0.001885

SEX2:SEX2.PE passed 0.4185 0.002010

SEX1.units passed 0.1573 0.000416

SEX2.units passed 0.1372 0.000380

> HPDinterval(model6$VCV)

lower upper

SEX1:SEX1.animal 0.02117354 0.1923723

SEX2:SEX1.animal -0.13258885 0.1871918

SEX1:SEX2.animal -0.13258885 0.1871918

SEX2:SEX2.animal 0.04519912 0.2512716

SEX1:SEX1.PE 0.02421394 0.5442394

SEX2:SEX1.PE 0.07339634 0.6102402

SEX1:SEX2.PE 0.07339634 0.6102402

SEX2:SEX2.PE 0.09301648 0.8584873

SEX1.units 0.10077967 0.2216915

SEX2.units 0.08079218 0.2021422

attr(,"Probability")

[1] 0.95

> vAf6 <- model6$VCV[,'SEX1:SEX1.animal']

> vRf6 <- model6$VCV[,'SEX1.units']

> VPEf6<- model6$VCV[,'SEX1:SEX1.PE']

>

> vAm6 <- model6$VCV[,'SEX2:SEX2.animal']

> vRm6 <- model6$VCV[,'SEX2.units']

> VPEm6<- model6$VCV[,'SEX2:SEX2.PE']

>

>

> mean(vAf6)

[1] 0.1034421

> HPDinterval(vAf6)

lower upper

var1 0.02117354 0.1923723

attr(,"Probability")

[1] 0.95

>

> mean(vRf6)

[1] 0.1572986

> HPDinterval(vRf6)

lower upper

var1 0.1007797 0.2216915

attr(,"Probability")

[1] 0.95

>

> mean(VPEf6)

[1] 0.2522814

> HPDinterval(VPEf6)

lower upper

var1 0.02421394 0.5442394

attr(,"Probability")

[1] 0.95

>

>

> mean(vAm6)

[1] 0.1467329

> HPDinterval(vAm6)

lower upper

var1 0.04519912 0.2512716

attr(,"Probability")

[1] 0.95

>

> mean(vRm6)

[1] 0.1371597

> HPDinterval(vRm6)

lower upper

var1 0.08079218 0.2021422

attr(,"Probability")

[1] 0.95

>

> mean(VPEm6)

[1] 0.4184998

> HPDinterval(VPEm6)

lower upper

var1 0.09301648 0.8584873

attr(,"Probability")

[1] 0.95

>

> heritF6<- vAf6/(vAf6 + vRf6 + VPEf6)

> mean(heritF6)

[1] 0.2116822

> HPDinterval(heritF6)

lower upper

var1 0.03669916 0.4040983

attr(,"Probability")

[1] 0.95

>

> heritM6<- vAm6/(vAm6 + vRm6 + VPEm6)

> mean(heritM6)

[1] 0.2217062

> HPDinterval(heritM6)

lower upper

var1 0.06020066 0.4000761

attr(,"Probability")

[1] 0.95

>

> CVAf6<- sqrt(model6$VCV[,'SEX1:SEX1.animal'])/3.31

> mean(CVAf6)

[1] 0.09471562

> HPDinterval(CVAf6)

lower upper

var1 0.05004728 0.1357495

attr(,"Probability")

[1] 0.95

>

> If6<- model6$VCV[,'SEX1:SEX1.animal']/ 10.94

> mean(If6)

[1] 0.009455399

> HPDinterval(If6)

lower upper

var1 0.001935424 0.01758431

attr(,"Probability")

[1] 0.95

>

> CVRf6<- sqrt(model6$VCV[,'SEX1.units'])/ 3.31

> mean(CVRf6)

[1] 0.1192335

> HPDinterval(CVRf6)

lower upper

var1 0.09740165 0.1435672

attr(,"Probability")

[1] 0.95

>

>

> CVPEf6<- sqrt(model6$VCV[,'SEX1:SEX1.PE'])/ 3.31

> mean(CVPEf6)

[1] 0.1456748

> HPDinterval(CVPEf6)

lower upper

var1 0.06641901 0.2328743

attr(,"Probability")

[1] 0.95

>

> CVAm6<- sqrt(model6$VCV[,'SEX2:SEX2.animal'])/ 4.04

> mean(CVAm6)

[1] 0.09318092

> HPDinterval(CVAm6)

lower upper

var1 0.05717131 0.1266764

attr(,"Probability")

[1] 0.95

>

> Im6<- model6$VCV[,'SEX2:SEX2.animal']/ 16.351

> mean(Im6)

[1] 0.008973939

> HPDinterval(Im6)

lower upper

var1 0.002764303 0.01536736

attr(,"Probability")

[1] 0.95

>

> CVRm6<- sqrt(model6$VCV[,'SEX2.units'])/ 4.04

> mean(CVRm6)

[1] 0.09105702

> HPDinterval(CVRm6)

lower upper

var1 0.07182374 0.1124034

attr(,"Probability")

[1] 0.95

>

> CVPEm6<- sqrt(model6$VCV[,'SEX2:SEX2.PE'])/ 4.04

> mean(CVPEm6)

[1] 0.1552049

> HPDinterval(CVPEm6)

lower upper

var1 0.08416832 0.2350039

attr(,"Probability")

[1] 0.95

>

> herit.diff6<- heritF6 - heritM6

> mean(herit.diff6)

[1] -0.01002397

> HPDinterval(herit.diff6)

lower upper

var1 -0.244208 0.232969

attr(,"Probability")

[1] 0.95

>

> CVA.diff6 <- CVAf6 - CVAm6

> mean(CVA.diff6)

[1] 0.001534705

> HPDinterval(CVA.diff6)

lower upper

var1 -0.05093662 0.05485204

attr(,"Probability")

[1] 0.95

>

> I.diff6 <- If6 - Im6

> mean(I.diff6)

[1] 0.0004814604

> HPDinterval(I.diff6)

lower upper

var1 -0.009238993 0.01015227

attr(,"Probability")

[1] 0.95

>

> CVR.diff6 <- CVRf6 - CVRm6

> mean(CVR.diff6)

[1] 0.0281765

> HPDinterval(CVR.diff6)

lower upper

var1 -0.003205897 0.05843546

attr(,"Probability")

[1] 0.95

>

>

> CVPE.diff6<- CVPEf6 - CVPEm6

> mean(CVPE.diff6)

[1] -0.009530083

> HPDinterval(CVPE.diff6)

lower upper

var1 -0.0961375 0.07459036

attr(,"Probability")

[1] 0.95

>

> vf6 <- sapply(1:nrow(model6[["Sol"]]), function(i) {var(predict(model6, it = i)) })

>

> mean(vf6)

[1] 0.006560115

>

> heritF6.1<- vAf6/(vAf6 + vRf6 + VPEf6 + vf6)

> mean(heritF6.1)

[1] 0.208474

> HPDinterval(heritF6.1)

lower upper

var1 0.03901163 0.3990986

attr(,"Probability")

[1] 0.95

>

> heritM6.1<- vAm6/(vAm6 + vRm6 + VPEm6 + vf6)

> mean(heritM6.1)

[1] 0.2196751

> HPDinterval(heritM6.1)

lower upper

var1 0.05922245 0.3963933

attr(,"Probability")

[1] 0.95

>

> herit.diff6.1<- heritF6.1 - heritM6.1

> mean(herit.diff6.1)

[1] -0.008804898

> HPDinterval(herit.diff6.1)

lower upper

var1 -0.2486454 0.2301229

attr(,"Probability")

[1] 0.95

>

> corr.gen2 <- model6$VCV[, 'SEX1:SEX2.animal']/sqrt((model6$VCV[,'SEX1:SEX1.animal']*model6$VCV[,'SEX2:SEX2.animal']))

> corr.gen2 <- model6$VCV[, 'SEX1:SEX2.animal']/sqrt((model6$VCV[,'SEX1:SEX1.animal']*model6$VCV[,'SEX2:SEX2.animal']))

> mean(corr.gen2)

[1] 0.6028411

> HPDinterval(corr.gen2)

lower upper

var1 -0.9739552 0.998888

attr(,"Probability")

[1] 0.95

### Prior 2

**prior2.1 <- list(R=list(V=diag(2), nu=0.02), G=list(G1=list(V=diag(2), nu=0.2, alpha.mu=c(0,0),alpha.V=diag(2)*1000), G2=list(V=diag(2), nu=0.2, alpha.mu=c(0,0),alpha.V=diag(2)*1000)))**

**model6 <- MCMCglmm(NS~SEX, random=~us(SEX):animal + us(SEX):PE, rcov=~idh(SEX):units, prior=prior2.1, pedigree=NA_ped, data=Data5, nitt=5000000, burnin=150000, thin=100)**

Iterations = 150001:4999901

Thinning interval = 100

Sample size = 48500

DIC: 361.2763

G-structure: ~us(SEX):animal

post.mean l-95% CI u-95% CI eff.samp

SEX1:SEX1.animal 0.10641 0.01868 0.1994 48500

SEX2:SEX1.animal 0.03843 -0.12884 0.1668 9801

SEX1:SEX2.animal 0.03843 -0.12884 0.1668 9801

SEX2:SEX2.animal 0.15112 0.05049 0.2612 49279

~us(SEX):PE

post.mean l-95% CI u-95% CI eff.samp

SEX1:SEX1.PE 0.2245 0.01619 0.4992 36278

SEX2:SEX1.PE 0.2740 0.04656 0.5722 40000

SEX1:SEX2.PE 0.2740 0.04656 0.5722 40000

SEX2:SEX2.PE 0.4302 0.09180 0.8865 48500

R-structure: ~idh(SEX):units

post.mean l-95% CI u-95% CI eff.samp

SEX1.units 0.1563 0.09981 0.2217 45556

SEX2.units 0.1353 0.08075 0.1999 48114

Location effects: NS ~ SEX

post.mean l-95% CI u-95% CI eff.samp pMCMC

(Intercept) 0.64088 0.52411 0.75907 40865 <2e-05 ***

SEX2 0.13726 -0.03875 0.31879 48500 0.133

---

Signif. codes: 0 ‘***’ 0.001 ‘**’ 0.01 ‘*’ 0.05 ‘.’ 0.1 ‘ ’ 1

> posterior.mode(model6$Sol[, "SEX2"])

var1

0.1495168

> plot(model6$Sol)

> plot(model6$VCV)

Hit <Return> to see next plot: autocorr.diag(model6$Sol)

Hit <Return> to see next plot: autocorr.diag(model6$VCV)

Hit <Return> to see next plot: effectiveSize(model6$Sol)

> effectiveSize(model6$VCV)

SEX1:SEX1.animal SEX2:SEX1.animal SEX1:SEX2.animal SEX2:SEX2.animal SEX1:SEX1.PE SEX2:SEX1.PE SEX1:SEX2.PE

48500.000 9801.098 9801.098 49279.462 36278.056 39999.695 39999.695

SEX2:SEX2.PE SEX1.units SEX2.units

48500.000 45556.073 48114.393

> heidel.diag(model6$VCV)

Stationarity start p-value

test iteration

SEX1:SEX1.animal passed 1 0.6609

SEX2:SEX1.animal passed 1 0.5731

SEX1:SEX2.animal passed 1 0.5731

SEX2:SEX2.animal passed 1 0.5205

SEX1:SEX1.PE passed 1 0.7959

SEX2:SEX1.PE passed 1 0.3402

SEX1:SEX2.PE passed 1 0.3402

SEX2:SEX2.PE passed 1 0.0981

SEX1.units passed 1 0.6496

SEX2.units passed 1 0.3767

Halfwidth Mean Halfwidth

test

SEX1:SEX1.animal passed 0.1064 0.000408

SEX2:SEX1.animal passed 0.0384 0.001598

SEX1:SEX2.animal passed 0.0384 0.001598

SEX2:SEX2.animal passed 0.1511 0.000477

SEX1:SEX1.PE passed 0.2245 0.001457

SEX2:SEX1.PE passed 0.2740 0.001454

SEX1:SEX2.PE passed 0.2740 0.001454

SEX2:SEX2.PE passed 0.4302 0.002050

SEX1.units passed 0.1563 0.000295

SEX2.units passed 0.1353 0.000287

> HPDinterval(model6$VCV)

lower upper

SEX1:SEX1.animal 0.01868411 0.1993555

SEX2:SEX1.animal -0.12884293 0.1668214

SEX1:SEX2.animal -0.12884293 0.1668214

SEX2:SEX2.animal 0.05048521 0.2612299

SEX1:SEX1.PE 0.01619193 0.4992347

SEX2:SEX1.PE 0.04656462 0.5722489

SEX1:SEX2.PE 0.04656462 0.5722489

SEX2:SEX2.PE 0.09180220 0.8864875

SEX1.units 0.09981289 0.2216783

SEX2.units 0.08075111 0.1999104

attr(,"Probability")

[1] 0.95

> vAf6 <- model6$VCV[,'SEX1:SEX1.animal']

> vRf6 <- model6$VCV[,'SEX1.units']

> VPEf6<- model6$VCV[,'SEX1:SEX1.PE']

>

> vAm6 <- model6$VCV[,'SEX2:SEX2.animal']

> vRm6 <- model6$VCV[,'SEX2.units']

> VPEm6<- model6$VCV[,'SEX2:SEX2.PE']

>

>

> mean(vAf6)

[1] 0.1064083

> HPDinterval(vAf6)

lower upper

var1 0.01868411 0.1993555

attr(,"Probability")

[1] 0.95

>

> mean(vRf6)

[1] 0.15634

> HPDinterval(vRf6)

lower upper

var1 0.09981289 0.2216783

attr(,"Probability")

[1] 0.95

>

> mean(VPEf6)

[1] 0.2244846

> HPDinterval(VPEf6)

lower upper

var1 0.01619193 0.4992347

attr(,"Probability")

[1] 0.95

>

>

> mean(vAm6)

[1] 0.1511163

> HPDinterval(vAm6)

lower upper

var1 0.05048521 0.2612299

attr(,"Probability")

[1] 0.95

>

> mean(vRm6)

[1] 0.1353141

> HPDinterval(vRm6)

lower upper

var1 0.08075111 0.1999104

attr(,"Probability")

[1] 0.95

>

> mean(VPEm6)

[1] 0.4301526

> HPDinterval(VPEm6)

lower upper

var1 0.0918022 0.8864875

attr(,"Probability")

[1] 0.95

>

> heritF6<- vAf6/(vAf6 + vRf6 + VPEf6)

> mean(heritF6)

[1] 0.2301559

> HPDinterval(heritF6)

lower upper

var1 0.03560636 0.4486823

attr(,"Probability")

[1] 0.95

>

> heritM6<- vAm6/(vAm6 + vRm6 + VPEm6)

> mean(heritM6)

[1] 0.2248311

> HPDinterval(heritM6)

lower upper

var1 0.06137741 0.4079719

attr(,"Probability")

[1] 0.95

>

> CVAf6<- sqrt(model6$VCV[,'SEX1:SEX1.animal'])/3.31

> mean(CVAf6)

[1] 0.09593211

> HPDinterval(CVAf6)

lower upper

var1 0.05112612 0.1405327

attr(,"Probability")

[1] 0.95

>

> If6<- model6$VCV[,'SEX1:SEX1.animal']/ 10.94

> mean(If6)

[1] 0.009726537

> HPDinterval(If6)

lower upper

var1 0.001707871 0.01822262

attr(,"Probability")

[1] 0.95

>

> CVRf6<- sqrt(model6$VCV[,'SEX1.units'])/ 3.31

> mean(CVRf6)

[1] 0.1188455

> HPDinterval(CVRf6)

lower upper

var1 0.09596208 0.1427239

attr(,"Probability")

[1] 0.95

>

>

> CVPEf6<- sqrt(model6$VCV[,'SEX1:SEX1.PE'])/ 3.31

> mean(CVPEf6)

[1] 0.1366333

> HPDinterval(CVPEf6)

lower upper

var1 0.05597812 0.2225369

attr(,"Probability")

[1] 0.95

>

> CVAm6<- sqrt(model6$VCV[,'SEX2:SEX2.animal'])/ 4.04

> mean(CVAm6)

[1] 0.09458488

> HPDinterval(CVAm6)

lower upper

var1 0.05924231 0.1288994

attr(,"Probability")

[1] 0.95

>

> Im6<- model6$VCV[,'SEX2:SEX2.animal']/ 16.351

> mean(Im6)

[1] 0.009242023

> HPDinterval(Im6)

lower upper

var1 0.003087592 0.01597639

attr(,"Probability")

[1] 0.95

>

> CVRm6<- sqrt(model6$VCV[,'SEX2.units'])/ 4.04

> mean(CVRm6)

[1] 0.09044639

> HPDinterval(CVRm6)

lower upper

var1 0.07110304 0.1113286

attr(,"Probability")

[1] 0.95

>

> CVPEm6<- sqrt(model6$VCV[,'SEX2:SEX2.PE'])/ 4.04

> mean(CVPEm6)

[1] 0.157228

> HPDinterval(CVPEm6)

lower upper

var1 0.08431113 0.2390593

attr(,"Probability")

[1] 0.95

>

> herit.diff6<- heritF6 - heritM6

> mean(herit.diff6)

[1] 0.005324823

> HPDinterval(herit.diff6)

lower upper

var1 -0.2518896 0.2741803

attr(,"Probability")

[1] 0.95

>

> CVA.diff6 <- CVAf6 - CVAm6

> mean(CVA.diff6)

[1] 0.001347233

> HPDinterval(CVA.diff6)

lower upper

var1 -0.05513515 0.05666707

attr(,"Probability")

[1] 0.95

>

> I.diff6 <- If6 - Im6

> mean(I.diff6)

[1] 0.0004845138

> HPDinterval(I.diff6)

lower upper

var1 -0.009809475 0.01085374

attr(,"Probability")

[1] 0.95

>

> CVR.diff6 <- CVRf6 - CVRm6

> mean(CVR.diff6)

[1] 0.02839908

> HPDinterval(CVR.diff6)

lower upper

var1 -0.002830939 0.06025962

attr(,"Probability")

[1] 0.95

>

>

> CVPE.diff6<- CVPEf6 - CVPEm6

> mean(CVPE.diff6)

[1] -0.02059471

> HPDinterval(CVPE.diff6)

lower upper

var1 -0.1132001 0.07033191

attr(,"Probability")

[1] 0.95

>

> vf6 <- sapply(1:nrow(model6[["Sol"]]), function(i) {var(predict(model6, it = i)) })

> mean(vf6)

[1] 0.00675993

>

> heritF6.1<- vAf6/(vAf6 + vRf6 + VPEf6 + vf6)

> mean(heritF6.1)

[1] 0.2263986

> HPDinterval(heritF6.1)

lower upper

var1 0.03652964 0.4416101

attr(,"Probability")

[1] 0.95

>

> heritM6.1<- vAm6/(vAm6 + vRm6 + VPEm6 + vf6)

> mean(heritM6.1)

[1] 0.2227395

> HPDinterval(heritM6.1)

lower upper

var1 0.0613431 0.4055537

attr(,"Probability")

[1] 0.95

>

>

> corr.gen2 <- model6$VCV[, 'SEX1:SEX2.animal']/sqrt((model6$VCV[,'SEX1:SEX1.animal']*model6$VCV[,'SEX2:SEX2.animal']))

> mean(corr.gen2)

[1] 0.3102093

> HPDinterval(corr.gen2)

lower upper

var1 -0.8940907 1

attr(,"Probability")

[1] 0.95

# Data

## Aggression

# SEX: 1 = female, 2 = male, AGG: aggressiveness score, Idf: contest ID, PE: maternal effect (mother ID)/common environment (egg-sac ID)

animal dam sire SEX AGG Idf PE

1 NA NA 1 NA NA NA

1 NA NA 1 NA NA NA

4 NA NA 1 11 1 NA

4 NA NA 1 3 2 NA

6 NA NA 1 NA NA NA

6 NA NA 1 NA NA NA

8 NA NA 1 0 3 NA

8 NA NA 1 0 14 NA

14 NA NA 1 3 4 NA

14 NA NA 1 0 9 NA

19 NA NA 1 0 5 NA

19 NA NA 1 0 10 NA

20 NA NA 1 2 6 NA

20 NA NA 1 3 29 NA

21 NA NA 1 3 28 NA

21 NA NA 1 5 42 NA

23 NA NA 1 0 35 NA

23 NA NA 1 6 45 NA

24 NA NA 1 1 1 NA

24 NA NA 1 1 24 NA

25 NA NA 1 3 2 NA

25 NA NA 1 0 7 NA

25 NA NA 1 2 41 NA

28 NA NA 1 3 8 NA

28 NA NA 1 1 46 NA

30 NA NA 1 0 26 NA

30 NA NA 1 1 36 NA

34 NA NA 1 6 4 NA

34 NA NA 1 0 9 NA

37 NA NA 1 1 37 NA

37 NA NA 1 0 44 NA

38 NA NA 1 0 7 NA

38 NA NA 1 0 41 NA

40 NA NA 1 3 5 NA

40 NA NA 1 2 10 NA

41 NA NA 1 3 36 NA

41 NA NA 1 3 43 NA

42 NA NA 1 1 38 NA

42 NA NA 1 4 39 NA

43 NA NA 1 0 11 NA

43 NA NA 1 0 15 NA

45 NA NA 1 10 12 NA

45 NA NA 1 6 32 NA

46 NA NA 1 NA NA NA

46 NA NA 1 NA NA NA

47 NA NA 1 15 13 NA

47 NA NA 1 11 22 NA

49 NA NA 1 0 3 NA

49 NA NA 1 0 14 NA

53 NA NA 1 3 11 NA

53 NA NA 1 2 15 NA

56 NA NA 1 NA NA NA

56 NA NA 1 NA NA NA

57 NA NA 1 3 16 NA

57 NA NA 1 4 34 NA

59 NA NA 1 0 17 NA

59 NA NA 1 1 40 NA

61 NA NA 1 0 12 NA

61 NA NA 1 0 18 NA

62 NA NA 1 6 19 NA

62 NA NA 1 4 30 NA

64 NA NA 1 0 20 NA

64 NA NA 1 0 33 NA

65 NA NA 1 NA NA NA

65 NA NA 1 NA NA NA

66 NA NA 1 1 21 NA

66 NA NA 1 1 23 NA

71 NA NA 1 NA NA NA

71 NA NA 1 NA NA NA

72 NA NA 1 16 13 NA

72 NA NA 1 13 22 NA

73 NA NA 1 NA NA NA

73 NA NA 1 NA NA NA

74 NA NA 1 NA NA NA

74 NA NA 1 NA NA NA

77 NA NA 1 1 21 NA

77 NA NA 1 1 23 NA

78 NA NA 1 2 8 NA

78 NA NA 1 5 24 NA

79 NA NA 1 0 25 NA

79 NA NA 1 1 31 NA

82 NA NA 1 0 26 NA

82 NA NA 1 0 36 NA

84 NA NA 1 NA NA NA

84 NA NA 1 NA NA NA

86 NA NA 1 0 44 NA

86 NA NA 1 NA NA NA

88 NA NA 1 0 20 NA

88 NA NA 1 0 27 NA

89 NA NA 1 2 38 NA

89 NA NA 1 2 39 NA

90 NA NA 1 0 35 NA

90 NA NA 1 0 45 NA

91 NA NA 1 4 28 NA

91 NA NA 1 7 42 NA

92 NA NA 1 0 6 NA

92 NA NA 1 0 29 NA

94 NA NA 1 0 17 NA

94 NA NA 1 0 40 NA

96 NA NA 1 2 19 NA

96 NA NA 1 1 30 NA

97 NA NA 1 0 25 NA

97 NA NA 1 0 31 NA

98 NA NA 1 0 18 NA

98 NA NA 1 9 32 NA

100 NA NA 1 1 27 NA

100 NA NA 1 0 33 NA

101 NA NA 1 5 16 NA

101 NA NA 1 3 34 NA

120 6 105 1 1 120 6

121 6 105 1 0 121 6

122 6 105 1 1 122 6

126 8 108 1 0 126 8

127 8 108 1 0 127 8

128 8 108 1 5 128 8

132 14 27 1 0,5 132 14

133 14 27 1 0,5 133 14

134 14 27 1 0 134 14

138 20 36 1 2 138 20

139 20 36 1 2 139 20

140 20 36 1 4 140 20

144 30 63 1 1 144 30

145 30 63 1 1 145 30

146 30 63 1 1,5 146 30

150 34 102 1 7 150 34

151 34 102 1 0,5 151 34

152 34 102 1 0 152 34

156 40 50 1 0 156 40

157 40 50 1 0 157 40

158 40 50 1 2 158 40

162 41 76 1 3 162 41

163 41 76 1 5 163 41

164 41 76 1 9 164 41

168 49 102 1 0 168 49

169 49 102 1 0 169 49

170 49 102 1 0 170 49

174 61 60 1 0 174 61

175 61 60 1 0 175 61

176 61 60 1 0 176 61

180 62 55 1 1,5 180 62

181 62 55 1 2 181 62

182 62 55 1 0 182 62

186 79 31 1 0 186 79

187 79 31 1 2 187 79

188 79 31 1 1 188 79

192 84 102 1 0 192 84

193 84 102 1 0 193 84

194 84 102 1 1 194 84

198 88 31 1 34 198 88

199 88 31 1 1 199 88

200 88 31 1 0 200 88

204 91 48 1 0 204 91

205 91 48 1 0 205 91

206 91 48 1 NA 206 91

210 98 44 1 0,5 210 98

211 98 44 1 0 211 98

212 98 44 1 1 212 98

216 100 95 1 0,5 216 100

217 100 95 1 0,5 217 100

218 100 95 1 0 218 100

222 101 83 1 7 222 101

223 101 83 1 3 223 101

224 101 83 1 0 224 101

2 NA NA 2 12 75 NA

2 NA NA 2 22 86 NA

3 NA NA 2 1 50 NA

3 NA NA 2 46 82 NA

5 NA NA 2 8 67 NA

5 NA NA 2 13 68 NA

7 NA NA 2 5 51 NA

7 NA NA 2 24 52 NA

26 NA NA 2 3 53 NA

26 NA NA 2 6 66 NA

27 NA NA 2 3 54 NA

27 NA NA 2 7 73 NA

29 NA NA 2 8 87 NA

29 NA NA 2 1 89 NA

31 NA NA 2 2 63 NA

31 NA NA 2 2 77 NA

32 NA NA 2 2 79 NA

32 NA NA 2 15 80 NA

33 NA NA 2 7 57 NA

33 NA NA 2 5 78 NA

35 NA NA 2 0 90 NA

35 NA NA 2 2 79 NA

36 NA NA 2 9 55 NA

36 NA NA 2 15 56 NA

44 NA NA 2 8 57 NA

44 NA NA 2 12 74 NA

48 NA NA 2 14 58 NA

48 NA NA 2 16 74 NA

50 NA NA 2 6 81 NA

50 NA NA 2 7 88 NA

51 NA NA 2 47 91 NA

51 NA NA 2 30 81 NA

55 NA NA 2 32 61 NA

55 NA NA 2 36 62 NA

58 NA NA 2 6 88 NA

58 NA NA 2 1 92 NA

60 NA NA 2 7 59 NA

60 NA NA 2 18 83 NA

63 NA NA 2 2 59 NA

63 NA NA 2 34 60 NA

69 NA NA 2 11 83 NA

69 NA NA 2 8 84 NA

70 NA NA 2 12 61 NA

70 NA NA 2 12 62 NA

75 NA NA 2 16 51 NA

75 NA NA 2 25 73 NA

76 NA NA 2 4 63 NA

76 NA NA 2 2 84 NA

80 NA NA 2 22 58 NA

80 NA NA 2 22 75 NA

81 NA NA 2 0 54 NA

81 NA NA 2 0 85 NA

83 NA NA 2 51 60 NA

83 NA NA 2 76 82 NA

85 NA NA 2 4 77 NA

85 NA NA 2 5 78 NA

87 NA NA 2 2 85 NA

87 NA NA 2 6 80 NA

95 NA NA 2 1 50 NA

95 NA NA 2 4 85 NA

99 NA NA 2 94 52 NA

99 NA NA 2 6 86 NA

102 NA NA 2 2 71 NA

102 NA NA 2 3 72 NA

103 NA NA 2 2 64 NA

103 NA NA 2 3 65 NA

104 NA NA 2 3 55 NA

104 NA NA 2 5 56 NA

105 NA NA 2 2 69 NA

105 NA NA 2 4 70 NA

106 NA NA 2 2 53 NA

106 NA NA 2 1 64 NA

107 NA NA 2 9 65 NA

107 NA NA 2 15 66 NA

108 NA NA 2 4 67 NA

108 NA NA 2 3 68 NA

109 NA NA 2 1 69 NA

109 NA NA 2 3 70 NA

110 NA NA 2 NA NA NA

110 NA NA 2 NA NA NA

111 NA NA 2 7 71 NA

111 NA NA 2 5 72 NA

123 6 105 2 0 152 6

124 6 105 2 0 150 6

125 6 105 2 3 151 6

129 8 108 2 7 160 8

130 8 108 2 0 150 8

131 8 108 2 NA NA 8

135 14 27 2 6 152 14

136 14 27 2 31 158 14

137 14 27 2 10 159 14

141 20 36 2 3 156 20

142 20 36 2 18 157 20

143 20 36 2 NA NA 20

147 30 63 2 16 155 30

148 30 63 2 8 154 30

149 30 63 2 7 153 30

153 34 102 2 16 155 34

154 34 102 2 7 161 34

155 34 102 2 36 162 34

159 40 50 2 13 163 40

160 40 50 2 9 156 40

161 40 50 2 NA NA 40

165 41 76 2 33 164 41

166 41 76 2 7 153 41

167 41 76 2 7 165 41

171 49 102 2 NA NA 49

172 49 102 2 NA NA 49

173 49 102 2 NA NA 49

177 61 60 2 23 166 61

178 61 60 2 14 165 61

179 61 60 2 1 167 61

183 62 55 2 43 168 62

184 62 55 2 7 169 62

185 62 55 2 38 166 62

189 79 31 2 35 170 79

190 79 31 2 7 171 79

191 79 31 2 7 172 79

195 84 102 2 20 164 84

196 84 102 2 10 168 84

197 84 102 2 16 173 84

201 88 31 2 1 163 88

202 88 31 2 0 169 88

203 88 31 2 NA NA 88

207 91 48 2 34 171 91

208 91 48 2 43 158 91

209 91 48 2 4 161 91

213 98 44 2 1 151 98

214 98 44 2 23 173 98

215 98 44 2 36 170 98

219 100 95 2 4 159 100

220 100 95 2 32 174 100

221 100 95 2 NA NA 100

225 101 83 2 15 160 101

226 101 83 2 58 172 101

227 101 83 2 34 154 101

## Activity

# SEX: 1 = female, 2 = male, NS: log(latency to stop walking + 1), PE: maternal effect (mother ID)/common environment (egg-sac ID)

animal dam sire SEX NS PE

1 NA NA 1 1,041392685 NA

1 NA NA 1 0,653212514 NA

2 NA NA 2 1,616685521 NA

2 NA NA 2 0,771587481 NA

3 NA NA 2 0,418301291 NA

3 NA NA 2 0,444044796 NA

4 NA NA 1 0,789580712 NA

4 NA NA 1 0,810904281 NA

5 NA NA 2 0,851258349 NA

5 NA NA 2 0,595496222 NA

6 NA NA 1 0,67669361 NA

6 NA NA 1 0 NA

7 NA NA 2 0,8876173 NA

7 NA NA 2 0,477121255 NA

8 NA NA 1 0,481442629 NA

8 NA NA 1 0,51851394 NA

14 NA NA 1 1,797475288 NA

14 NA NA 1 0,913283902 NA

19 NA NA 1 1,369215857 NA

19 NA NA 1 1,064457989 NA

20 NA NA 1 1,393048466 NA

20 NA NA 1 0,670245853 NA

21 NA NA 1 0,87909588 NA

21 NA NA 1 0,977723605 NA

23 NA NA 1 0,531478917 NA

23 NA NA 1 0,342422681 NA

24 NA NA 1 0,625312451 NA

24 NA NA 1 1,239299479 NA

25 NA NA 1 0,696356389 NA

25 NA NA 1 0,722633923 NA

26 NA NA 2 0,309630167 NA

26 NA NA 2 0,408239965 NA

27 NA NA 2 1,548389418 NA

27 NA NA 2 1,703549298 NA

28 NA NA 1 1,69434191 NA

28 NA NA 1 0,827369273 NA

29 NA NA 2 0,899820502 NA

29 NA NA 2 0,679427897 NA

30 NA NA 1 0 NA

30 NA NA 1 0,673941999 NA

31 NA NA 2 1,176958981 NA

31 NA NA 2 0,693726949 NA

32 NA NA 2 1,859378504 NA

32 NA NA 2 1,330413773 NA

33 NA NA 2 1,812512284 NA

33 NA NA 2 1,379668034 NA

34 NA NA 1 0,764176132 NA

34 NA NA 1 0,418301291 NA

35 NA NA 2 0,802089258 NA

35 NA NA 2 0,665580991 NA

36 NA NA 2 1,356981401 NA

36 NA NA 2 1,039017322 NA

37 NA NA 1 0,846955325 NA

37 NA NA 1 1,096910013 NA

38 NA NA 1 0 NA

38 NA NA 1 0 NA

40 NA NA 1 0,864511081 NA

40 NA NA 1 0,911157609 NA

41 NA NA 1 0,382017043 NA

41 NA NA 1 0 NA

42 NA NA 1 0,342422681 NA

42 NA NA 1 0,428134794 NA

43 NA NA 1 0,725094521 NA

43 NA NA 1 0,352182518 NA

44 NA NA 2 1,355259906 NA

44 NA NA 2 1,62930764 NA

45 NA NA 1 1,21005085 NA

45 NA NA 1 1,123851641 NA

46 NA NA 1 0,588831726 NA

46 NA NA 1 0,782472624 NA

47 NA NA 1 0,866287339 NA

47 NA NA 1 0,612783857 NA

48 NA NA 2 0,875061263 NA

48 NA NA 2 0,743509765 NA

49 NA NA 1 0,658964843 NA

49 NA NA 1 0,759667845 NA

50 NA NA 2 0,915399835 NA

50 NA NA 2 1,213252052 NA

51 NA NA 2 1,024074987 NA

51 NA NA 2 0,960946196 NA

53 NA NA 1 1,722962809 NA

53 NA NA 1 0,729974286 NA

55 NA NA 2 0,352182518 NA

55 NA NA 2 1,101059355 NA

56 NA NA 1 0,864511081 NA

56 NA NA 1 0,787460475 NA

57 NA NA 1 0 NA

57 NA NA 1 0 NA

58 NA NA 2 0 NA

58 NA NA 2 0 NA

59 NA NA 1 0,495544338 NA

59 NA NA 1 0 NA

60 NA NA 2 0,813580989 NA

60 NA NA 2 0 NA

61 NA NA 1 0,63748973 NA

61 NA NA 1 0,612783857 NA

62 NA NA 1 0,536558443 NA

62 NA NA 1 1,009450896 NA

63 NA NA 2 1,81517913 NA

63 NA NA 2 0,727541257 NA

64 NA NA 1 1,755951041 NA

64 NA NA 1 1,481155871 NA

65 NA NA 1 0 NA

65 NA NA 1 0,357934847 NA

66 NA NA 1 0 NA

66 NA NA 1 0,555094449 NA

69 NA NA 2 0,764922985 NA

69 NA NA 2 0,875061263 NA

70 NA NA 2 0,477121255 NA

70 NA NA 2 0,459392488 NA

71 NA NA 1 1,77633791 NA

71 NA NA 1 1,003891166 NA

72 NA NA 1 0,532754379 NA

72 NA NA 1 0,646403726 NA

73 NA NA 1 0,511883361 NA

73 NA NA 1 0 NA

74 NA NA 1 0,727541257 NA

74 NA NA 1 0,361727836 NA

75 NA NA 2 0,281033367 NA

75 NA NA 2 1,298197867 NA

76 NA NA 2 0,804139432 NA

76 NA NA 2 1,042575512 NA

77 NA NA 1 1,8831502 NA

77 NA NA 1 0,977723605 NA

78 NA NA 1 0 NA

78 NA NA 1 0 NA

79 NA NA 1 0,413299764 NA

79 NA NA 1 0,382017043 NA

80 NA NA 2 0,591064607 NA

80 NA NA 2 0,622214023 NA

81 NA NA 2 0 NA

81 NA NA 2 0 NA

82 NA NA 1 1,984257202 NA

82 NA NA 1 0,720159303 NA

83 NA NA 2 1,091666958 NA

83 NA NA 2 0,966141733 NA

84 NA NA 1 0 NA

84 NA NA 1 0 NA

85 NA NA 2 0,7355989 NA

85 NA NA 2 0,397940009 NA

86 NA NA 1 1,031408464 NA

86 NA NA 1 0,598790507 NA

87 NA NA 2 1,682325619 NA

87 NA NA 2 1,640481437 NA

88 NA NA 1 0,790988475 NA

88 NA NA 1 0,540329475 NA

89 NA NA 1 0,614897216 NA

89 NA NA 1 0 NA

90 NA NA 1 0,987666265 NA

90 NA NA 1 0,595496222 NA

91 NA NA 1 0,823474229 NA

91 NA NA 1 0,380211242 NA

92 NA NA 1 0,519827994 NA

92 NA NA 1 0,715167358 NA

94 NA NA 1 0,936010796 NA

94 NA NA 1 0,496929648 NA

95 NA NA 2 1,336859821 NA

95 NA NA 2 0,597695186 NA

96 NA NA 1 0 NA

96 NA NA 1 0,692846919 NA

97 NA NA 1 0 NA

97 NA NA 1 0,942008053 NA

98 NA NA 1 0,804820679 NA

98 NA NA 1 0 NA

99 NA NA 2 1,324282455 NA

99 NA NA 2 1,732634968 NA

100 NA NA 1 1,01494035 NA

100 NA NA 1 0,481442629 NA

101 NA NA 1 0,489958479 NA

101 NA NA 1 0,62838893 NA

102 NA NA 2 0,64738297 NA

102 NA NA 2 0,352182518 NA

103 NA NA 2 0 NA

103 NA NA 2 0,320146286 NA

104 NA NA 2 1,122215878 NA

104 NA NA 2 0,640481437 NA

105 NA NA 2 1,546419267 NA

105 NA NA 2 1,068927612 NA

106 NA NA 2 0 NA

106 NA NA 2 0 NA

107 NA NA 2 0,419955748 NA

107 NA NA 2 0,439332694 NA

108 NA NA 2 1,192009593 NA

108 NA NA 2 0,7355989 NA

109 NA NA 2 0,494154594 NA

109 NA NA 2 0 NA

110 NA NA 2 1,364175633 NA

110 NA NA 2 0 NA

111 NA NA 2 0,866287339 NA

111 NA NA 2 0,46834733 NA

120 6 105 1 0 6

121 6 105 1 0,644438589 6

122 6 105 1 0,505149978 6

123 6 105 2 0,567026366 6

124 6 105 2 0,892651034 6

125 6 105 2 0 6

126 8 108 1 0 8

127 8 108 1 0,477121255 8

128 8 108 1 0 8

129 8 108 2 0 8

130 8 108 2 0,517195898 8

131 8 108 2 NA 8

132 14 27 1 0,301029996 14

133 14 27 1 0 14

134 14 27 1 0 14

135 14 27 2 1,819543936 14

136 14 27 2 0 14

137 14 27 2 0 14

138 20 36 1 0 20

139 20 36 1 2,478566496 20

140 20 36 1 0 20

141 20 36 2 0 20

142 20 36 2 0 20

143 20 36 2 NA 20

144 30 63 1 0 30

145 30 63 1 1,849480837 30

146 30 63 1 0 30

147 30 63 2 0 30

148 30 63 2 0 30

149 30 63 2 0 30

150 34 102 1 0 34

151 34 102 1 0 34

152 34 102 1 0 34

153 34 102 2 0,176091259 34

154 34 102 2 0 34

155 34 102 2 0 34

156 40 50 1 0 40

157 40 50 1 0 40

158 40 50 1 0 40

159 40 50 2 0 40

160 40 50 2 0 40

161 40 50 2 NA 40

162 41 76 1 0,477121255 41

163 41 76 1 0 41

164 41 76 1 0 41

165 41 76 2 0 41

166 41 76 2 0 41

167 41 76 2 0 41

168 49 102 1 1,833402129 49

169 49 102 1 0 49

170 49 102 1 0 49

171 49 102 2 NA 49

172 49 102 2 NA 49

173 49 102 2 NA 49

174 61 60 1 1,387567779 61

175 61 60 1 0 61

176 61 60 1 0 61

177 61 60 2 0 61

178 61 60 2 0,924795996 61

179 61 60 2 0 61

180 62 55 1 0 62

181 62 55 1 0 62

182 62 55 1 0 62

183 62 55 2 0 62

184 62 55 2 1,294686624 62

185 62 55 2 0 62

186 79 31 1 0 79

187 79 31 1 0 79

188 79 31 1 0 79

189 79 31 2 0 79

190 79 31 2 1,293583513 79

191 79 31 2 0 79

192 84 102 1 0 84

193 84 102 1 0 84

194 84 102 1 0,301029996 84

195 84 102 2 0,411619706 84

196 84 102 2 0 84

197 84 102 2 1,278753601 84

198 88 31 1 0 88

199 88 31 1 0 88

200 88 31 1 0 88

201 88 31 2 0 88

202 88 31 2 0 88

203 88 31 2 NA 88

204 91 48 1 0 91

205 91 48 1 0 91

206 91 48 1 NA 91

207 91 48 2 0 91

208 91 48 2 0 91

209 91 48 2 0 91

210 98 44 1 0 98

211 98 44 1 0 98

212 98 44 1 0 98

213 98 44 2 0 98

214 98 44 2 0 98

215 98 44 2 0 98

216 100 95 1 0 100

217 100 95 1 0,709269961 100

218 100 95 1 0 100

219 100 95 2 0 100

220 100 95 2 0,837588438 100

221 100 95 2 NA 100

222 101 83 1 0 101

223 101 83 1 0 101

224 101 83 1 0 101

225 101 83 2 0 101

226 101 83 2 0 101

227 101 83 2 0 101
